# Optimization of Inhibitors of *Mycobacterium tuberculosis* Pantothenate Synthetase Based on Group Efficiency Analysis

**DOI:** 10.1002/cmdc.201500414

**Published:** 2015-10-21

**Authors:** Alvin W. Hung, H. Leonardo Silvestre, Shijun Wen, Guillaume P. C. George, Jennifer Boland, Tom L. Blundell, Alessio Ciulli, Chris Abell

**Affiliations:** ^1^ Department of ChemistryUniversity of CambridgeLensfield RdCambridgeCB2 1EWUK; ^2^ Department of BiochemistryUniversity of Cambridge80 Tennis Court RdCambridgeCB2 1GAUK; ^3^ Experimental Therapeutic CentreA-STAR11 Biopolis WaySingapore138667Singapore; ^4^ Division of Biological Chemistry & Drug DiscoveryCollege of Life SciencesJames Black CentreUniversity of DundeeDow StreetDundeeDD1 5EHUK

**Keywords:** drug design, fragment-based screening, group efficiency, *Mycobacterium tuberculosis*, pantothenate synthetase

## Abstract

Ligand efficiency has proven to be a valuable concept for optimization of leads in the early stages of drug design. Taking this one step further, group efficiency (GE) evaluates the binding efficiency of each appendage of a molecule, further fine‐tuning the drug design process. Here, GE analysis is used to systematically improve the potency of inhibitors of *Mycobacterium tuberculosis* pantothenate synthetase, an important target in tuberculosis therapy. Binding efficiencies were found to be distributed unevenly within a lead molecule derived using a fragment‐based approach. Substitution of the less efficient parts of the molecule allowed systematic development of more potent compounds. This method of dissecting and analyzing different groups within a molecule offers a rational and general way of carrying out lead optimization, with potential broad application within drug discovery.

The concept of ligand efficiency (LE)^1^, ^2^, ^3^ has been used as an important guiding principle for lead optimization in early stage drug design. Subsequently, the idea of group efficiency (GE)^4^ was proposed, and this has been applied to identify hotpots on proteins and to analyze parts of a ligand that make important contributions to binding.^5^, ^6^, ^7^, ^8^, ^9^ Herein, we describe the use of GE to optimize a fragment‐derived inhibitor of *Mycobacterium tuberculosis* pantothenate synthetase, an attractive target for developing new drugs against tuberculosis.^10^, ^11^, ^12^

Pantothenate synthetase catalyzes the ATP‐dependent formation of an amide bond between pantoate and β‐alanine.^11^, ^12^ We have previously reported the identification of fragments **1** and **2** (see Schemes 1 and 2) from biophysical screens using thermal shift and NMR methods.^13^, ^14^ The stepwise growing of indole fragment **1** led to the generation of lead compound **5** (Scheme 1; see also, Figure S1 in the Supporting Information).^13^ In a parallel study, linking of fragments **1** and **2** afforded compounds **6**–**9** (Scheme 2; see also, Figure S2 in the Supporting Information).^**[**13, 15]^ Both fragment growing and linking approaches rapidly led to relatively potent inhibitors against pantothenate synthetase (**5**: *K*_D_=1.5 μm and **9**: *K*_D_=0.9 μm).

**Scheme 1:**
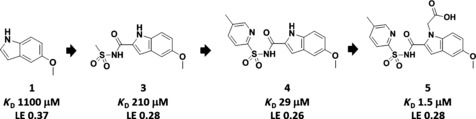
A fragment‐growing approach applied against *Mycobacterium tuberculosis* pantothenate synthetase, generating lead compound **5**.

**Scheme 2:**
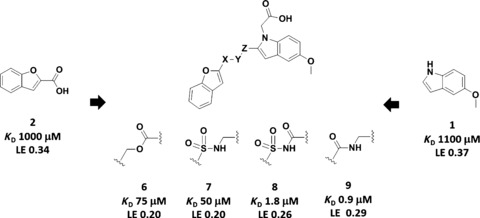
A fragment‐linking approach applied against *Mycobacterium tuberculosis* pantothenate synthetase generating lead compounds **6**–**9**. (X–Y–Z represents the approximate three‐atom length of the linker.)

Based solely on the *K*_D_ values and LE data obtained for compounds **5** and **8**, it was not obvious how to identify areas and vectors for further optimization of the compounds. Therefore, in an effort to improve binding potency while maintaining LE,^16^, ^17^ a GE approach was used to analyze compounds **5** and **8**. Following a similar Free–Wilson analysis^18^ as proposed by Saxty and co‐workers,^6^ compounds **5** and **8** were dissected into component parts, and the binding contributions (ΔΔ*G*) from these individual building blocks calculated. These data are summarized in Figure 1 (for detailed calculations of GE, see the section entitled “*Calculations for GE analysis*” in the Supporting Information). This GE analysis around **5** and **8** quickly revealed inefficient binding components within the molecules, suggesting straightforward approaches to fragment modifications without indiscriminately increasing the inhibitor size.

**Figure 1:**
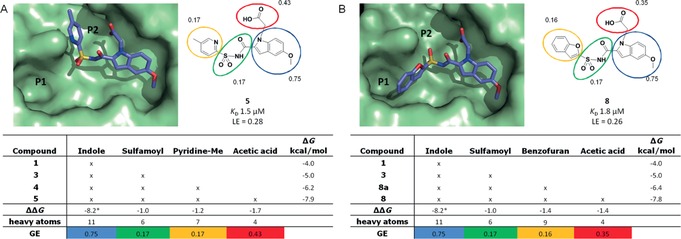
A) Group efficiency (GE) analysis of compound **5** estimates the contributions of the binding efficiencies from different functional groups and quickly reveals inefficient binding groups in the molecule for further optimization of potency. B) A similar GE analysis applied towards compound **8**. *ΔΔ*G*=Δ*G*−Δ*G*_rigid_, Δ*G*_rigid_=4.2 kcal mol^−1^.^19^ The cross‐sectional view of the X‐ray crystal structure of the active pocket of pantothenate synthetase is shown in green with inhibitors **5** and **8** bound. (The cross‐sectional view was generated by removing residues from one half of the active pocket of pantothenate synthetase using the DS visualizer software. The surface on the other half of the protein was generated using PyMol v.0.99^20^). Δ*G* values of the compounds were determined from titration experiments using ITC. The GE value is subsequently calculated by dividing the Δ*G* contribution from each group by the number of heavy atoms in the group.

As shown in Figure 1, the majority of the binding energy resides in the original indole fragment (GE=0.75). A similar observation has been observed in other fragment elaboration strategies^6^ and is mainly due to the high intrinsic binding energies required for fragments to be detected (Δ*G*=4.2 kcal mol^−1[19]^ is added to the binding of indole group **1** to compensate for the free energy associated with loss of fragment rigid‐body entropy during binding).

Similarly, the charged acetate side chain in both **5** and **8** also contributes significantly to the overall binding, possessing high GE values of 0.43 and 0.35, respectively. Conversely, GE analysis highlights the limited contribution to binding of the acyl sulfonamide, methyl pyridine and the benzofuran groups, all of which have calculated GE values of 0.16–0.17 (the GE of the sulfamoyl group is based on the *N*‐(methylsulfonyl)acetamide group).

Previous work on fragment growing and linking^13^ have shown that the acyl sulfonamide groups not only contributed to additional binding energy by forming additional hydrogen bonds between the sulfone oxygen and both the backbone amide of Met 40 and the side chain of His 47, but more importantly also served as an effective functional group for directing the correct vectors towards both the P1 and P2 pockets without clashing with the side of the active site formed by Met 40 (Figure S4 in the Supporting Information). Based on these observations, the initial optimization strategy was focused on replacement of the methyl pyridine and the benzofuran groups rather than the acyl sulfonamide linker in both compounds **5** and **8**.

Compounds were designed with the objective of optimizing interactions at the P1 or P2 sites of panthothenate synthetase. Consequently, 1‐ethyl‐3‐(3‐dimethylaminopropyl)‐carbodiimide (EDCI)‐mediated coupling reactions were employed to generate a series of indole acyl sulfonamide compounds (**10**–**19**; the syntheses are described in the Supporting Information).

The binding of compounds **10**–**19** against *M. tuberculosis* pantothenate synthetase was determined by isothermal titration calorimetry (ITC), and the structure–affinity relationship (SAR) results are summarized in Table 1; ITC binding data for all compounds are presented in the Supporting Information). Replacing the methyl pyridine/benzofuran groups in **5** and **8** generated a series of sub‐micromolar inhibitors (**10**–**14**).The substitution of the methyl pyridine ring (**5**) by a more electron‐rich toluene group (**10**: *K*_D_=340 nm, LE=0.32) resulted in approximately a fourfold improvement in affinity towards the enzyme. Compound **10** was the most ligand efficient compound tested. The GE value for the toluene group in **10** is 0.35—a marked improvement over the GE values of 0.17 and 0.16 for the methyl pyridine and benzofuran groups in **5** and **8**, respectively.

**Table 1:** Indole sulfonamide analogues **10**–**19** derived from SAR considerations around **5** and **8**.

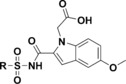										
Compd	R	*K*_D_ [μm]^[a]^	LE (GE)^[b]^	cLog *P* ^[c]^		Compd	R	*K*_D_ [μm]^[a]^	LE (GE)^[b]^	cLog *P* ^[c]^
**5**	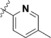	1.5	0.28 (0.17)	3.0		**14**	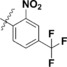	0.80	0.25 (0.15)	4.2
**8**	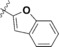	1.8	0.26 (0.16)	3.7		**15**	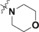	3.5	0.28 (0.17)	1.4
**10**	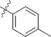	0.34	0.32 (0.35)	3.6		**16**	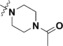	17	0.22 (0.01)	1.0
**11**	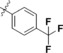	0.2	0.30 (0.27)	4.4		**17**	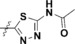	4	0.25 (0.17)	1.6
**12**	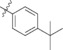	0.46	0.28 (0.22)	5.0		**18**	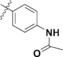	6	0.23 (0.07)	2.2
**13**	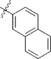	0.61	0.27 (0.21)	4.3		**19**	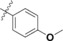	2	0.27 (0.17)	3.2

[a] *K*_D_ values were determined from titration experiments using ITC. [b] Ligand efficiency (LE) and group efficiency (GE) were calculated based on Δ*G* values derived from ITC and the number of heavy atoms associated with the corresponding groups/compounds. [c] cLog *P* values were derived from ChemDraw.

Gratifyingly, the addition of a bulkier and more electronegative trifluromethyl group to the indole sulfonamide core gave rise to **11**, the most potent compound of this series (*K*_D_=200 nm, LE=0.30, GE=0.27). Likewise, the installation of hydrophobic and larger groups like *tert*‐butylbenzene (**12**) and naphthalene (**13**) gave rise to compounds that bind to pantothenate synthetase with improved affinity (*K*_D_=460 nm and 610 nm, respectively) compared with parent compounds **5** and **8**. Interestingly, the more hydrophilic indole acyl sulfonamides **15**–**19** (cLog *P*=1.0–3.2) bind with lower affinities (*K*_D_=2–17 μm) compared with more lipophilic compounds **10**–**14** (cLog *P*=3.6–5.0).

This step of the optimization process was achieved in large part based on GE and SAR analysis without much consideration of structure. However, it did assume that the new compounds bind at the active site of pantothenate synthetase in a similar way to the original lead compounds, **5** and **8**. In order to establish this, the structures of the four most potent inhibitors (**10**–**13**) bound to the enzyme were solved using protein X‐ray crystallography (Figure 2).

**Figure 2:**
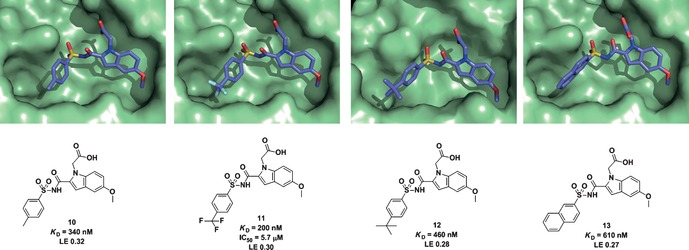
The X‐ray crystal structures of four of the most potent compounds (**10**–**13**) bound to *Mycobacterium tuberculosis* pantothenate synthetase (PDB code: 4MQ6, 4MUE, 4MUF, 4MUL, respectively). The ligands are shown as sticks with carbon atoms in light blue, nitrogen atoms in dark blue, oxygen atoms in red, and sulfur atoms in yellow. The cross‐sectional area of the active pocket of pantothenate synthetase is shown in green. All figures were generated and rendered with PyMOL v.0.99.^20^

The X‐ray crystal structures of **10**–**13** bound to pantothenate synthetase show binding at the active site, with a conserved binding mode for the indole sulfonamide fragment core. Less obviously, the substituted groups on all four compounds were seen to bind in the P1 pocket of the enzyme (see Figure 1 B). The P1 pocket binds the alkyl groups of the pantoate substrate and is primarily lipophilic, surrounded by the hydrophobic residues Pro 38, Met 40, Val 143, Leu 146 and Phe 157 (Figure S5 in the Supporting Information). In contrast, the P2 site binds the phosphates of ATP and is relatively hydrophilic. As can be seen in Figure 2, the binding orientations of the added groups are all similar, and no new hydrogen bonds are formed. The detailed binding interactions of the most potent compound (**11**) with the P1 pocket residues are shown in Figure S5 in the Supporting Information. In addition to binding assays and X‐ray crystallography studies, an inhibition study was carried out that demonstrated that compound **11** inhibits pantothenate synthetase with an IC_50_ value of 5.7 μm (see the Supporting Information).

The structural data on compounds **10**–**13** provided the impetus for further elaboration of the series, with a view to making a compound that probes more deeply into the P1 site. It was rationalized that the introduction of a methylene group between the aromatic and sulfonyl groups should allow the aromatic group to slide below Met 40 and push a *para* substituent to the back of the P1 pocket (Figure 3; for detailed binding interactions of **11** with the P1 pocket, see also Figure S5 in the Supporting Information). To test this hypothesis, compound **20** was synthesized, using a trifluoromethyl‐substituted benzylsulfonamide as a new coupling substrate.

**Figure 3:**
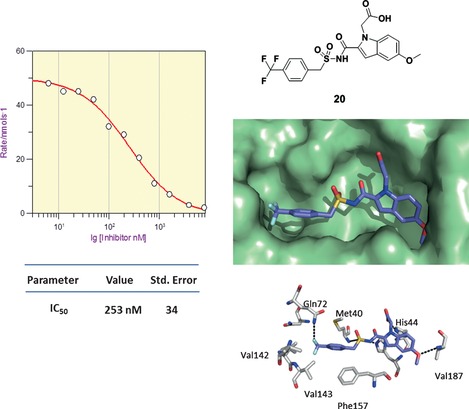
Compound **20** was found to inhibit *Mycobacterium tuberculosis* pantothenate synthetase with an IC_50_ value of 253 nm (LE=0.28 based on IC_50_). The X‐ray crystal structure of **20** bound to the active pocket of the enzyme shows the hydrophobic trifluoromethyl benzene group buried deep in the P1 site, surrounded by lipophilic residues Gln 72, Val 142 and Val 143 (PDB code: 4MUK).

The X‐ray crystal structure of **20** bound to *M. tuberculosis* pantothenate synthetase (Figure 3) showed the hoped for binding with the trifluoromethyl group picking up favorable hydrophobic interactions with Val 139, Val 142 and Val 143. Compound **20** was shown to inhibit the enzymatic reaction with a significantly improved IC_50_ value of 250 nm as compared with **11**. Furthermore, a cell‐based assay against *M. tuberculosis* showed on‐target inhibitory activity leading to cell death.^21^

As the use of fragment‐based methods expands, the need for subsequent lead optimization of fragment‐derived compounds becomes increasingly important. The work presented here demonstrates the use of GE analysis to critically and thoroughly examine the binding distribution of a lead compound and illustrates the practicality of applying GE analysis to modify parts of a molecule that are not making efficient contributions to binding. In this case, it led to the generation of a relatively potent and bactericidal inhibitor of *M. tuberculosis* pantothenate synthetase.

## Experimental Section

Syntheses and characterization of organic molecules, biochemical, X‐ray crystallography and isothermal titration calorimetry methods are described in the Supporting Information. Additionally, NMR spectra related to this publication are also available at the University of Cambridge data repository (www.repository.cam.ac.uk/handle/1810/249084).

Protein X‐ray crystallography structures of compounds **10**–**13** and **20** bound to *M. tuberculosis* pantothenate synthetase are available via the RCSB Protein Data Bank via PDB codes: 4MQ6 (**10**), 4MUE (**11**), 4MUF (**12**), 4MUL (**13**), and 4MUK (**20**).

## Supporting information

As a service to our authors and readers, this journal provides supporting information supplied by the authors. Such materials are peer reviewed and may be re‐organized for online delivery, but are not copy‐edited or typeset. Technical support issues arising from supporting information (other than missing files) should be addressed to the authors.

Supplementary

## References

[1] A. L. Hopkins , C. R. Groom , A. Alex , Drug Discovery Today 2004, 9, 430. 15109945 10.1016/S1359-6446(04)03069-7

[2] I. D. Kuntz , K. Chen , K. A. Sharp , P. A. Kollman , Proc. Natl. Acad. Sci. USA 1999, 96, 9997. 10468550 10.1073/pnas.96.18.9997 PMC17830

[3] A. L. Hopkins , G. M. Keseru , P. D. Leeson , D. C. Rees , C. H. Reynolds , Nat. Rev. Drug Discovery 2014, 13, 105. 24481311 10.1038/nrd4163

[4] M. L. Verdonk , D. C. Rees , ChemMedChem 2008, 3, 1179. 18651625 10.1002/cmdc.200800132

[5] A. Ciulli , G. Williams , A. G. Smith , T. L. Blundell , C. Abell , J. Med. Chem. 2006, 49, 4992. 16884311 10.1021/jm060490r

[6] G. Saxty , S. J. Woodhead , V. Berdini , T. G. Davies , M. L. Verdonk , P. G. Wyatt , R. G. Boyle , D. Barford , R. Downham , M. D. Garrett , R. A. Carr , J. Med. Chem. 2007, 50, 2293. 17451234 10.1021/jm070091b

[7] G. E. de Kloe , K. Retra , M. Geitmann , P. Källblad , T. Nahar , R. van Elk , A. B. Smit , J. E. van Muijlwijk Koezen , R. Leurs , H. Irth , U. H. Danielson , I. J. P. de Esch , J. Med. Chem. 2010, 53, 7192. 20828128 10.1021/jm100834y

[8] S. Barelier , J. Pons , O. Marcillat , J.-M. Lancelin , I. Krimm , J. Med. Chem. 2010, 53, 2577. 20192224 10.1021/jm100009z

[9] I. Van Molle , A. Thomann , D. L. Buckley , E. C. So , S. Lang , C. M. Crews , A. Ciulli , Chem. Biol. 2012, 19, 1300. 23102223 10.1016/j.chembiol.2012.08.015 PMC3551621

[10] V. K. Sambandamurthy , X. Wang , B. Chen , R. G. Russell , S. Derrick , F. M. Collins , S. L. Morris , W. R. Jacobs , Nat. Med. 2002, 8, 1171. 12219086 10.1038/nm765

[11] S. Wang , D. Eisenberg , Biochemistry 2006, 45, 1554. 16460002 10.1021/bi051873e

[12] R. Zheng , J. S. Blanchard , Biochemistry 2001, 40, 12904. 11669627 10.1021/bi011522+

[13] A. W. Hung , H. L. Silvestre , S. Wen , A. Ciulli , T. L. Blundell , C. Abell , Angew. Chem. Int. Ed. 2009, 48, 8452; 10.1002/anie.200903821 19780086

[14] H. L. Silvestre , T. L. Blundell , C. Abell , A. Ciulli , Proc. Natl. Acad. Sci. USA 2013, 110, 12984. 23872845 10.1073/pnas.1304045110 PMC3740835

[15] P. Sledz , H. L. Silvestre , A. W. Hung , A. Ciulli , T. L. Blundell , C. Abell , J. Am. Chem. Soc. 2010, 132, 4544. 20232910 10.1021/ja100595u PMC4441724

[16] P. D. Leeson , B. Springthorpe , Nat. Rev. Drug Discovery 2007, 6, 881. 17971784 10.1038/nrd2445

[17] M. M. Hann , MedChemComm 2011, 2, 349.

[18] S. M. Free , J. W. Wilson , J. Med. Chem. 1964, 7, 395. 14221113 10.1021/jm00334a001

[19] C. W. Murray , M. L. Verdonk , J. Comput.-Aided Mol. Des. 2002, 16, 741. 12650591 10.1023/a:1022446720849

[20] W. L. Delano, The Pymol Molecular Graphics Systems v.0.99, Delano Scientific, San Carlos, **2002**.

[21] G. L. Abrahams , A. Kumar , S. Savvi , A. W. Hung , S. Wen , C. Abell , C. E. Barry , D. R. Sherman , H. I. Boshoff , V. Mizrahi , Chem. Biol. 2012, 19, 844. 22840772 10.1016/j.chembiol.2012.05.020 PMC3421836

