# Supplementary material for: Optimization of Inhibitors of Mycobacterium tuberculosis Pantothenate Synthetase Based on Group Efficiency Analysis
Source: ChemMedChem. 2015 Oct 21;11(1):38–42. doi: 10.1002/cmdc.201500414 (PMC4949533; doi:10.1002/cmdc.201500414)
Supplement: Supplementary file 1 — Supplementary [file CMDC-11-38-s001.pdf]

CHEMMEDCHEM
Chemistry & Drug Discovery

Supporting Information

Optimization of Inhibitors of *Mycobacterium tuberculosis* Pantothenate Synthetase Based on Group Efficiency Analysis

Alvin W. Hung,[a] H. Leonardo Silvestre,[b] Shijun Wen,[a] Guillaume P. C. George,[a] Jennifer Boland,[a] Tom L. Blundell,[b] Alessio Ciulli,[a] and Chris Abell*[a]

cmdc_201500414_sm_miscellaneous_information.pdf

Supporting Information

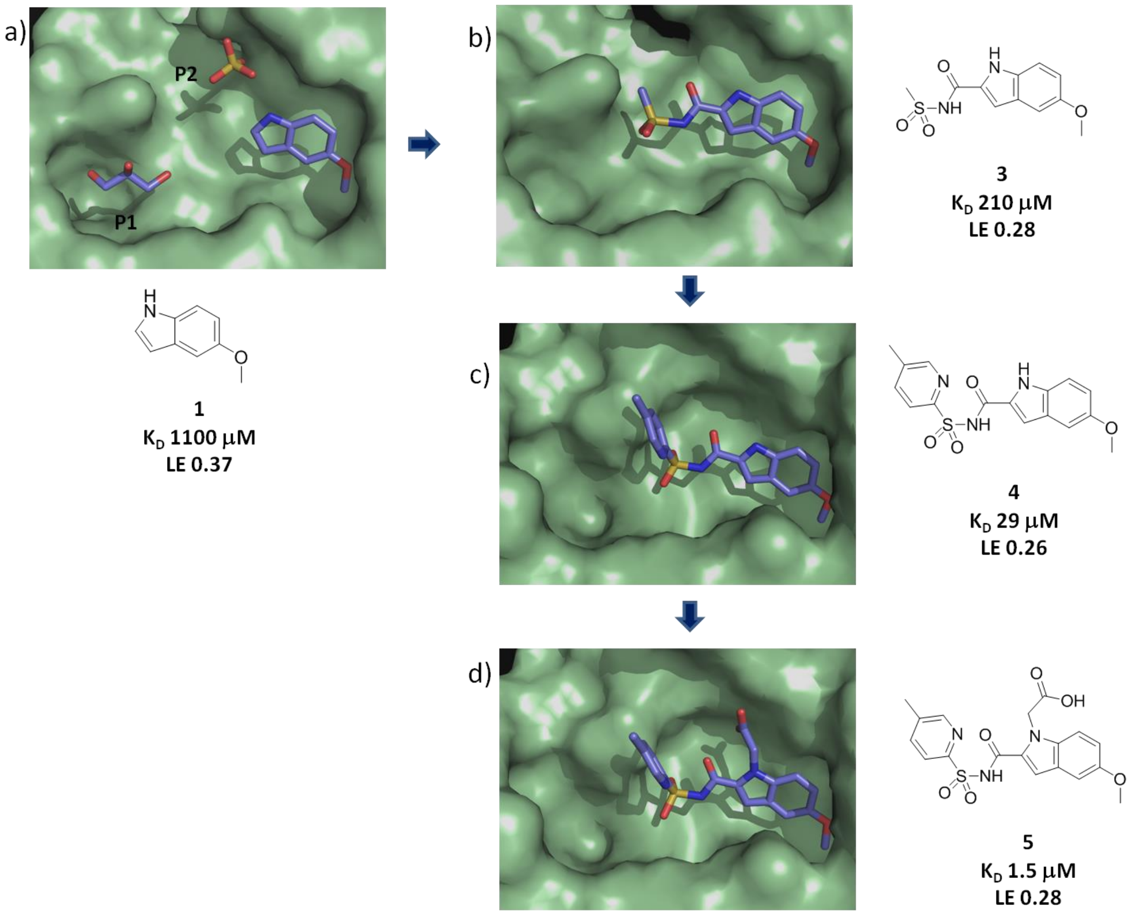

Figure S1. Fragment growing strategy applied to fragment **1** against *M.tuberculosis* pantothenate synthetase[1]. The X-ray crystal structures of compounds **1** – **5** binding at the active site of *M.tuberculosis* pantothenate synthetase show retention of the binding modes of the ligands, allowing GE analysis to be carried out. The cross-section of the active pocket of pantothenate synthetase is shown in green.

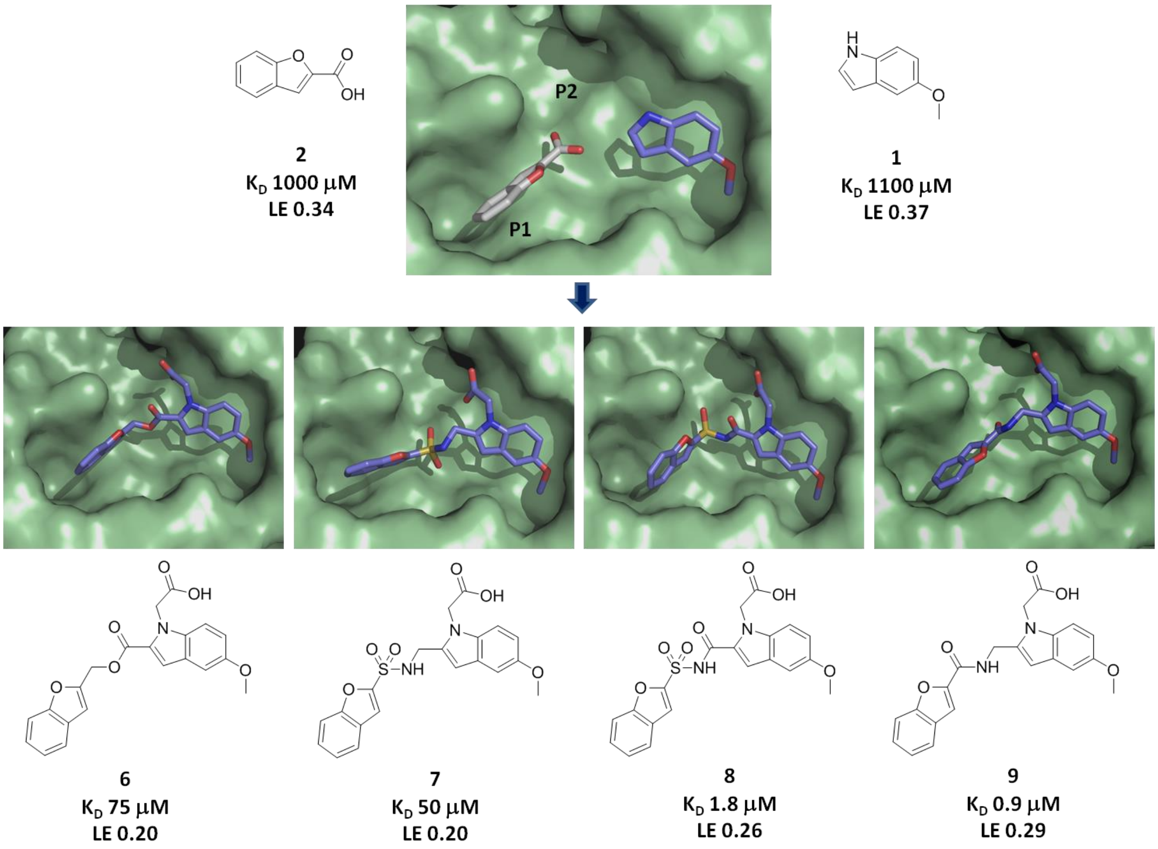

Figure S2. Fragment linking strategy applied to fragments **1** and **2** against *M.tuberculosis* pantothenate synthetase [1-2]. The cross-section of the active pocket of pantothenate synthetase is shown in green.

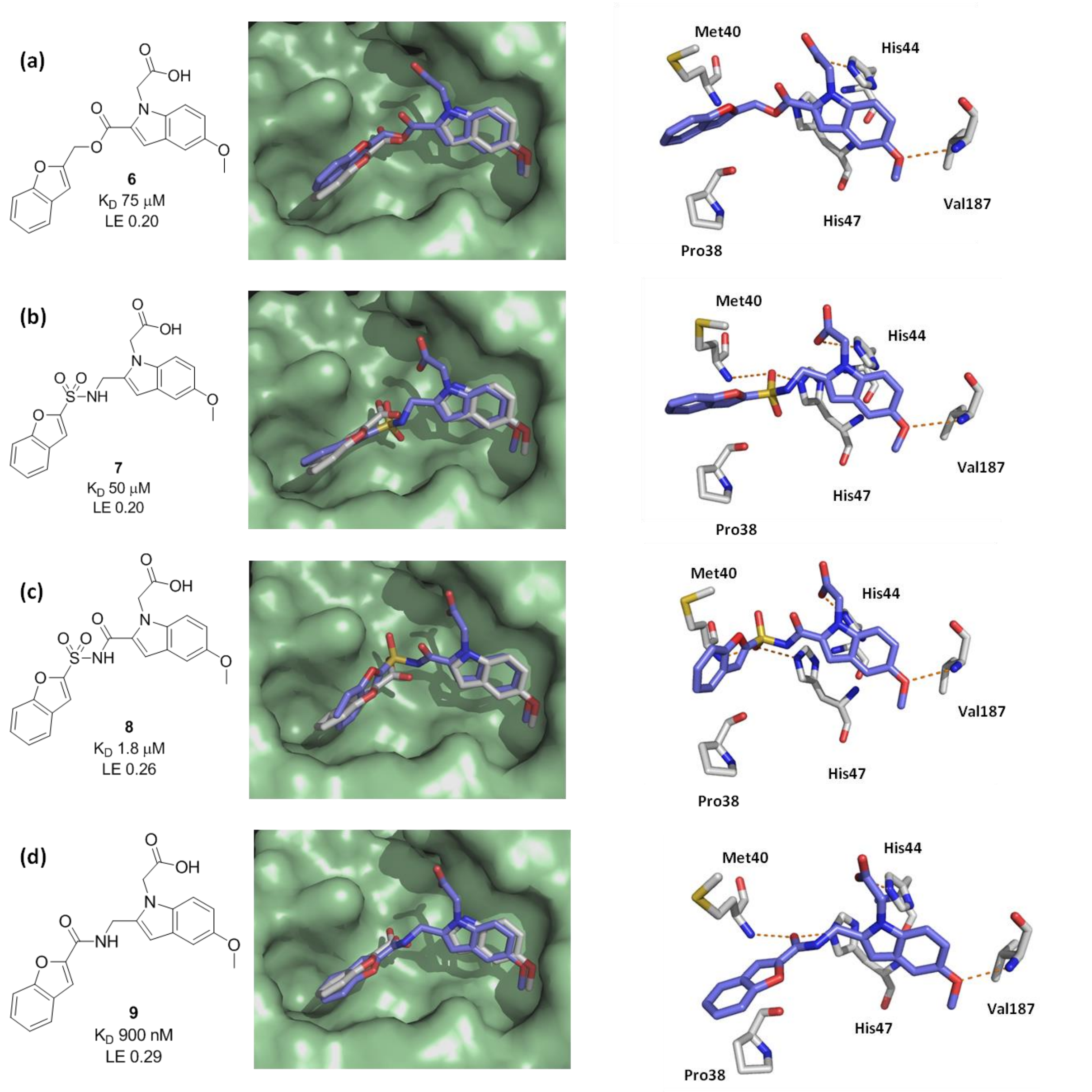

Figure S3. Overlay of the linked compounds with the original fragments binding against *M.tuberculosis* pantothenate synthetase. Detailed binding interactions of linked compounds with the active site residues are shown on the right. Carbon atoms for the original fragments **1** and **2** are in white, cross-section of the active pocket of pantothenate synthetase is shown in green.

__Calculations for Group Efficiency Analysis__

$$LE = -\Delta\Delta G \text{ / number of heavy atoms in group}$$

$$\Delta\Delta G = \Delta G_{group1} - \Delta G_{group2}$$

ΔG values were caculated based on K_d values obtained from ITC experiments.(SI Figures 7 – 12)

$$\Delta G = RT\,ln K_d$$

Where R = 1.987 cal.K^{-1}.mol^{-1} and T = 298 K

Compound	*Kd (mM)*	*-ΔG kcal/mol*	*NHA (total)*
**1**	1.1	4.03	11
**3**	0.21	5.01	18
**4**	0.03	6.17	24
**5**	0.0015	7.94	28

Compound	*Kd (mM)*	*-ΔG kcal/mol*	*NHA (total)*
**1**	1.1	4.03	11
**3**	0.21	5.01	18
**8a**	0.02	6.41	26
**8**	0.0018	7.83	30

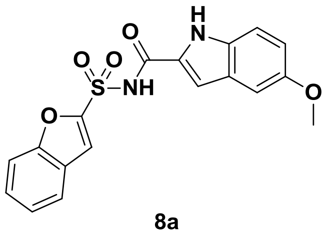

8a

Group Efficiency Analysis

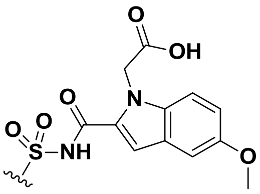

Based on **Figure 1B** of the paper, the theoretical contribution of binding for 2-(5-methoxy-2-(methylsulfonylcarbamoyl)-1H-indol-1-yl)acetic acid group above is -6.4 *kcal/mol*.

***ΔG kcal/mol** = -4 *kcal/mol* (indole) + -1.4 *kcal/mol* (acetic acid) + -1.0 *kcal/mol* (sulfamoyl) = - 6.4 kcal/mol.

Compound	***ΔG kcal/mol***	***-(ΔG kcal/mol - *ΔG kcal/mol)***	***Group***	***NHA***	***GE***
**10**	-8.82	2.42	toluene	7	0.35
**11**	-9.13	2.73	trifluromethylbenzene	10	0.27
**12**	-8.64	2.24	*tert*-butylbenzene	10	0.22
**13**	-8.47	2.07	napthalene	10	0.21
**14**	-8.31	1.91	nitrotrifluoromethylbenzene	13	0.15
**15**	-7.44	1.04	morpholine	6	0.17
**16**	-6.50	0.10	piperazine-amide	9	0.01
**17**	-7.36	1.0	thiadiazol-acetamide	9	0.1
**18**	-7.12	0.72	phenylacetamide	10	0.07
**19**	-7.77	1.37	methoxybenzene	8	0.17

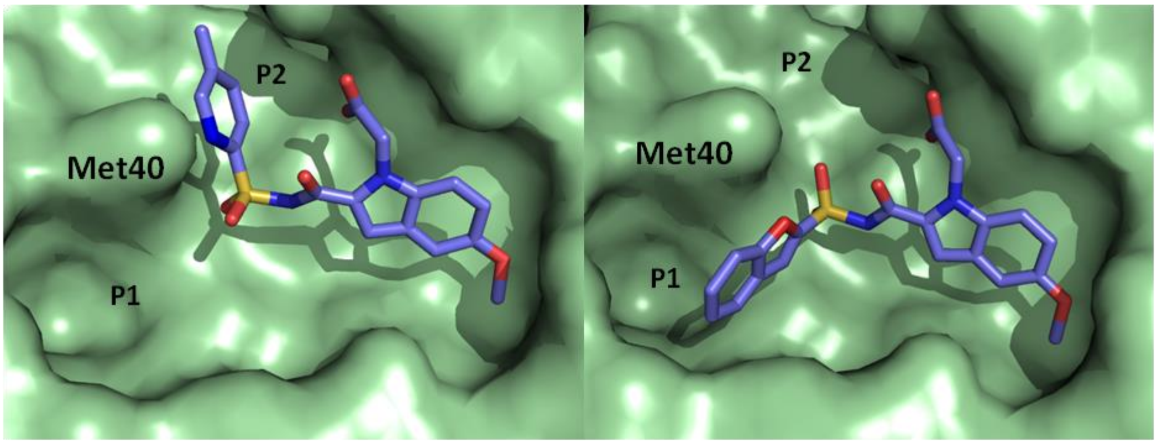

Figure S4. The acyl sulfonamide moiety direct groups to either the P1 or P2 pockets of *M.tuberculosis* Pts, avoiding clash against the protein wall comprising of Met40.

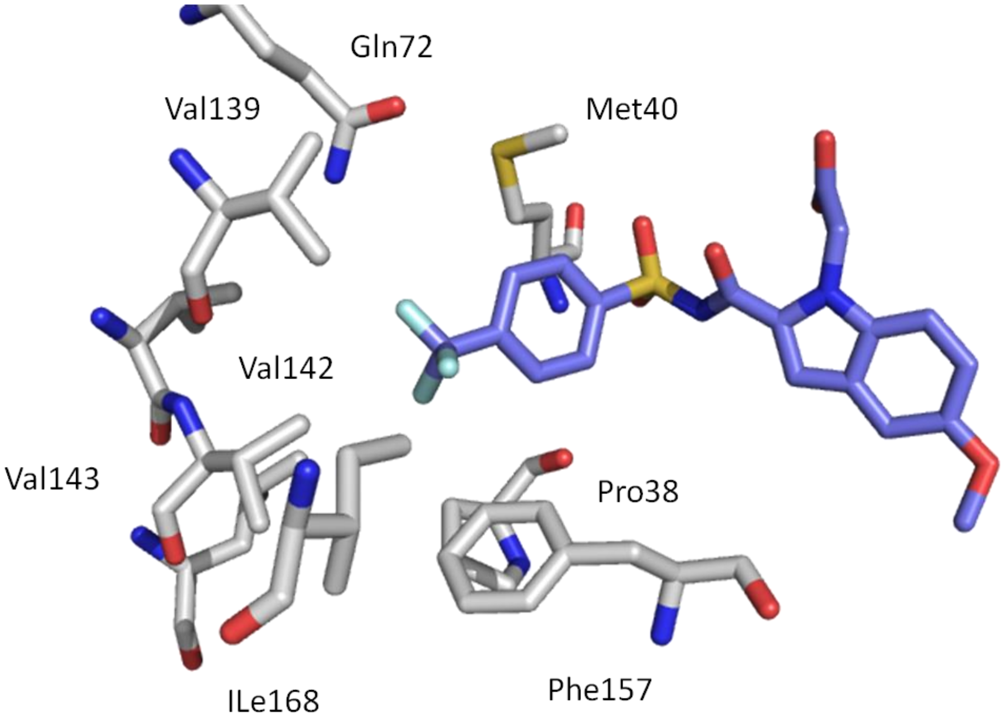

Figure S5. Detailed binding interactions of **11** with *M.tuberculosis* pantothenate synthetase at the P1 active site. Carbon atoms for the residues around the P1 pocket is shown in white.

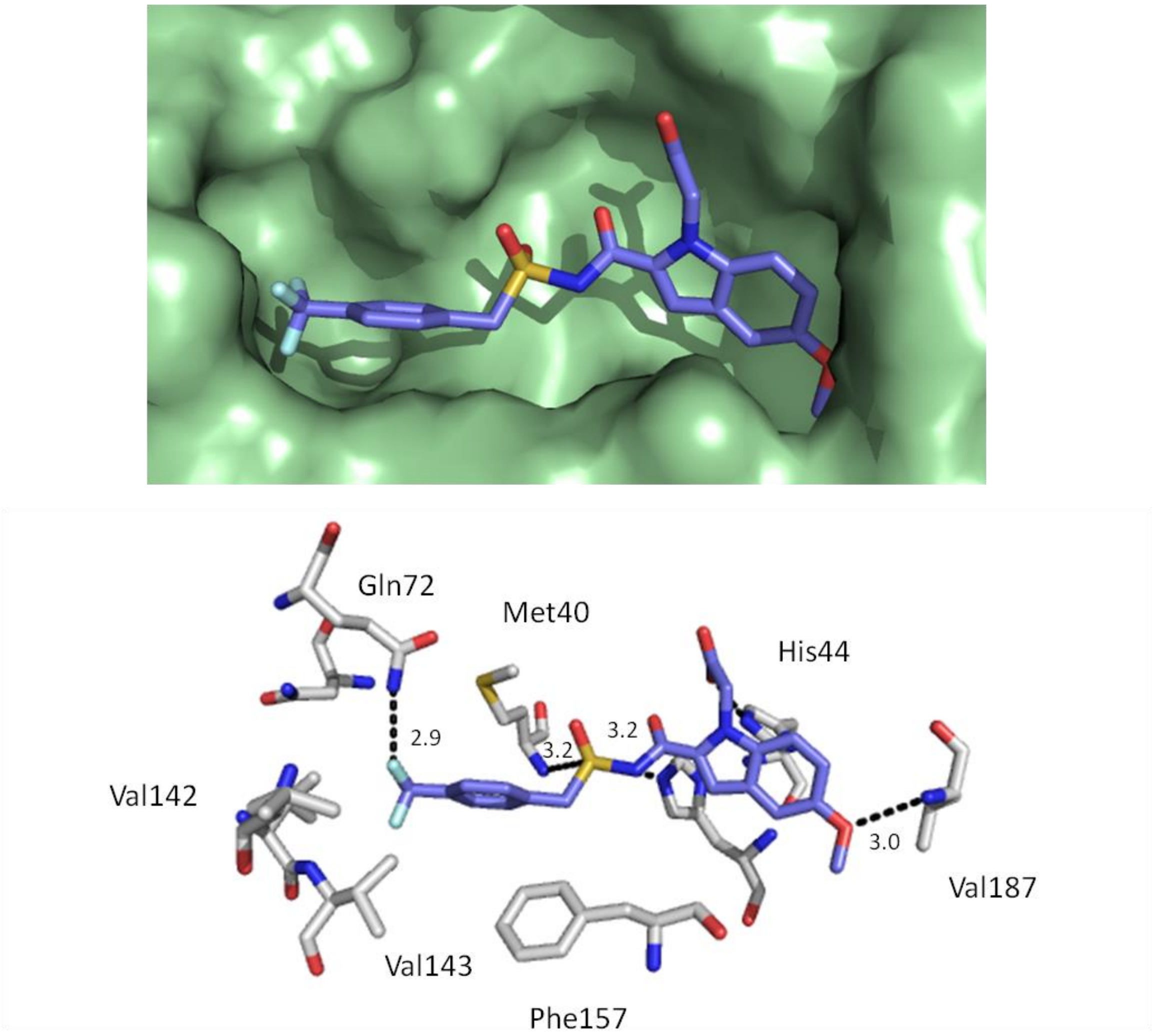

Figure S6. The detailed binding interactions of **20** at the active site of *M.tuberculosis* pantothenate synthetase. The extra methylene group is able to provide greater ligand flexibility and enables the trifluorobenzyl group to be poised deeper into the P1 pocket.

***Expression and purification of pantothenate* synthetase**[1] – The plasmid pET30a:*panC* encoding pantothenate synthetase from *M. tuberculosis* was kindly provided by Dr. Wang and Dr Eisenberg, University of California Los Angeles. This plasmid adds 44 amino acid residues to the *N*-terminus of the recombinant protein (MHHHHHHSSGLVPRGSGM KETAAAKFERQHMDSPDLGTDDDDKA–), including a 6×His tag and an enterokinase cleavage site. C41(DE3) competent cells transformed with the pET30a:*panC* plasmid were grown at 37 °C to an A_{600} of 0.6 in 2xYT medium containing 50 mg/mL kanamycin. After addition of IPTG (0.5 mM) the culture was grown for an additional 12 h at 30 °C. Cells (16 g from a 2 L culture) were suspended in 50 mL of 50 mM sodium phosphate, pH 8.0, 300 mM sodium chloride, 10 mM imidazole, 0.007% v/v β–mercaptoethanol and 1 mM phenylmethanesulphonylflouride protease inhibitor. Cells were lysed by incubating for 30 min at 25 ^{o}C with addition of 0.2 mg/ml of lysozyme, 10 mg/ml DNase and 10 mM magnesium chloride, followed by sonication for a total process time of 3 min. The cell lysate was applied to a Ni-NTA column (GE Healthcare), washed with 50 mM sodium phosphate, pH 8.0, 30 mM imidazole, 300 mM sodium chloride, and eluted with 250 mM imidazole. Fractions containing the desired protein were buffer-exchanged into 50 mM HEPES-HCl, pH 7.6, 50 mM sodium chloride, 5 mM magnesium chloride, using a HiPrep 26/10 Desalting column (GE Healthcare), to yield ~55 mg of His_6-PS from 1 L culture. The His-tagged protein was stored at -80 °C and used for biophysical studies and kinetic assays.

Crystallization, X-ray data collection, structure determination and refinement – For crystallographic studies, the His-tag was cleaved by enterokinase digestion as described previously[1]. Crystals of the untagged protein were grown by the hanging-drop vapour diffusion method at 20 °C. The well solution contained 11–14% w/v PEG3000, 100–150 mM lithium sulfate, 100 mM imidazole, pH 8.0, 2−4% v/v ethanol, 10% v/v glycerol and 20 mM magnesium chloride. Crystals were grown initially by mixing 1 μL of the protein with an equal volume of the well solution.

In order to facilitate the displacement of the sulfate bound at the active site, the crystals were transferred into 2 μL of a solution containing 14% PEG3000, 0.15 M LiCl, 0.1 M imidazole pH 8.0, and 4% ethanol prior to performing soaking experiments. Crystals were subsequently soaked overnight with 2 μL of a solution containing the ligands at concentrations ranging from 10 to 100 mM in 1–10 % v/v DMSO.

The crystals were cryoprotected in 30% glycerol and flash-frozen in liquid nitrogen for transportation. Data collection was carried at the Swiss Light Source PX III X06DA, at the European Synchrotron Radiation Facility, beam stations ID29, ID14-4, ID14-2 or at the Synchrotron Radiation Source, beam station 10.1 (Daresbury, United Kingdom). All derived data was indexed and scaled using either iMosflm and Scala or Denzo and Scalepack, respectively. Refinement was carried out using the graphical interface of the CCP4 suite, running Refmac 5.0[3]. Model building was done using COOT[4]. Data collection and refinement statistics for all the refined coordinate sets are presented in Supplementary Table 1.

Table S1. Crystallographic data collection and refinement statistics for **10 – 13**.

Data Collection	**10**	**11**	**12**	**13**
X-Ray Source	SLS, PXIII	ESRF, ID14-4	SLS, PX III	SLS, PX III
Space Group	$P2_1$	$P2_1$	$P2_1$	$P2_1$
Cell Parameters, Å (α=γ=90^0)				
a	48.54	48.64	48.73	48.72
b	70.90	70.44	71.06	70.86
c	81.61	81.90	81.69	81.83
β	99.36	99.6	99.46	99.42
Resolution range, Å (outer shell)	25.2 – 1.70 (1.79 – 1.70)	80.85 –2.06 (2.17 – 2.06)	44.6 – 2.50 (2.64 –2.50)	21.4 – 1.75 (1.84 – 1.75)
No. of unique reflections	60099	33903	19121	55392
Multiplicity	3.7	3.7	3.5	3.7
R_{merge} % (outer shell)	0.071 (0.580)	0.091 (0.532)	0.141 (0.595)	0.062 (0.586)
Average $I/\sigma(I)$	12.4	11.5	9.2	13.1
Completeness % (outer shell)	99.9 (99.8)	100 (100)	99.5 (99.4)	99.9 (99.9)
Mosaicity, ^{0}	0.6	0.7	0.6	0.6
Wilson B, $Å^2$	17.10	25.19	42.46	23.09
Refinement				
R_{cryst},	0.16	0.18	0.19	0.18
R_{free},	0.21	0.25	0.28	0.23
Number of reflections				
Working set	57043	32168	18138	52563
Test set	3033	1718	982	765
Ligands bound to active site				
Glycerol	0	0	0	0
Fragment/Inhibitor	3	3	2	2
Ethylene Glycol	0	8	0	0
PDB code	4MQ6	4MUE	4MUF	4MUL

Table S2. Crystallographic data collection and refinement statistics for **14-15, 17 and 19**.

Data Collection	**14**	**15**	**17**	**19**
X-Ray Source	SLS, PXIII	SLS, PXIII	SLS, PXIII	SLS, PXIII
Space Group	$P2_1$	$P2_1$	$P2_1$	$P2_1$
Cell Parameters, Å (α=γ=90^0)				
a	48.37	48.27	48.41	48.97
b	70.81	70.88	71.07	70.69
c	81.78	81.59	81.64	81.69
β	99.15	99.11	98.91	99.91
Resolution range, Å (outer shell)	25.22-1.57 (1.67-1.58)	21.96-1.54 (1.62-1.54)	28.61-1.72 (1.81-1.72)	32.36-2.10 (2.21-2.10)
No. of unique reflections	74634	78883	57664	32201
Multiplicity	3.7	3.8	3.7	5.6
R_{merge} % (outer shell)	0.063 (0.549)	0.119(0.453)	0.072(0.555)	0.189 (0.542)
Average $I/\sigma(I)$	13	9.4	12.8	10.4
Completeness % (outer shell)	99.9 (99.9)	98.2 (96.9)	100 (100)	100 (100)
Mosaicity, ^{0}		0.6	0.6	0.6
Wilson B, Å^2	18.80	17.95	20.77	24.61
Refinement				
R_{cryst},	0.16	0.19	0.17	0.19
R_{free},	0.20	0.24	0.21	0.25
Number of reflections				
Working set	72184	74922	55150	30552
Test set	3816	3956	2949	1631
Ligands bound to active site				
Glycerol	3	2	0	0
Fragment/Inhibitor	4	2	3	3
Ethylene Glycol	5	5	8	3
PDB code	4MUN	4MUG	4MUH	4MUI

Table S3. Crystallographic data collection and refinement statistics for **20**.

Data Collection	**20**
X-Ray Source	SLS, PXIII
Space Group	$P2_1$
Cell Parameters, Å (α=γ=90^0)	
a	48.62
b	70.91
c	81.81
β	99.5
Resolution range, Å (outer shell)	80.6-1.90 (2.0 – 1.90)
No. of unique reflections	41188
Multiplicity	3.6
R_{merge} % (outer shell)	0.07 (0.519)
Average $I/\sigma(I)$	13.5
Completeness % (outer shell)	95.1 (89.4)
Mosaicity, ^{0}	1.01
Wilson B, Å^2	22.79
Refinement	
R_{cryst},	0.18
R_{free},	0.23
Number of reflections	
Working set	39100
Test set	2088
Ligands bound to active site	
Glycerol	1
Fragment/Inhibitor	2
Ethylene Glycol	4
PDB code	4MUK

Isothermal titration calorimetry. ITC experiments were performed on either an ITC_{200} instruments from Microcal Inc. (GE Healthcare) at 25 ^{o}C. His_6-PS was buffered exchanged in 50 mM HEPES-HCl, pH 7.6 containing 50 mM sodium chloride and 5 mM magnesium chloride, and loaded into the ITC cell in concentrations of 50–100 μM with 5–10 % v/v DMSO solution. Ligands in concentrations of 0.5–15 mM (> 95% HPLC purity) were dissolved in the same buffer/DMSO solution and loaded into the syringe. Typically, on the ITC_{200}, 18 injections of 2.4 μl were performed over a period of 30 min with stirring at 1000 rpm. Results of the titrations for compounds **10 - 19** are shown in the figures below. The titration curves were plotted using Orgin 7 software provided by Microcal ITC_{200}. ΔG values were caluclated based on the K_a of the titration curves ($\Delta G = RT\,ln K_d$).

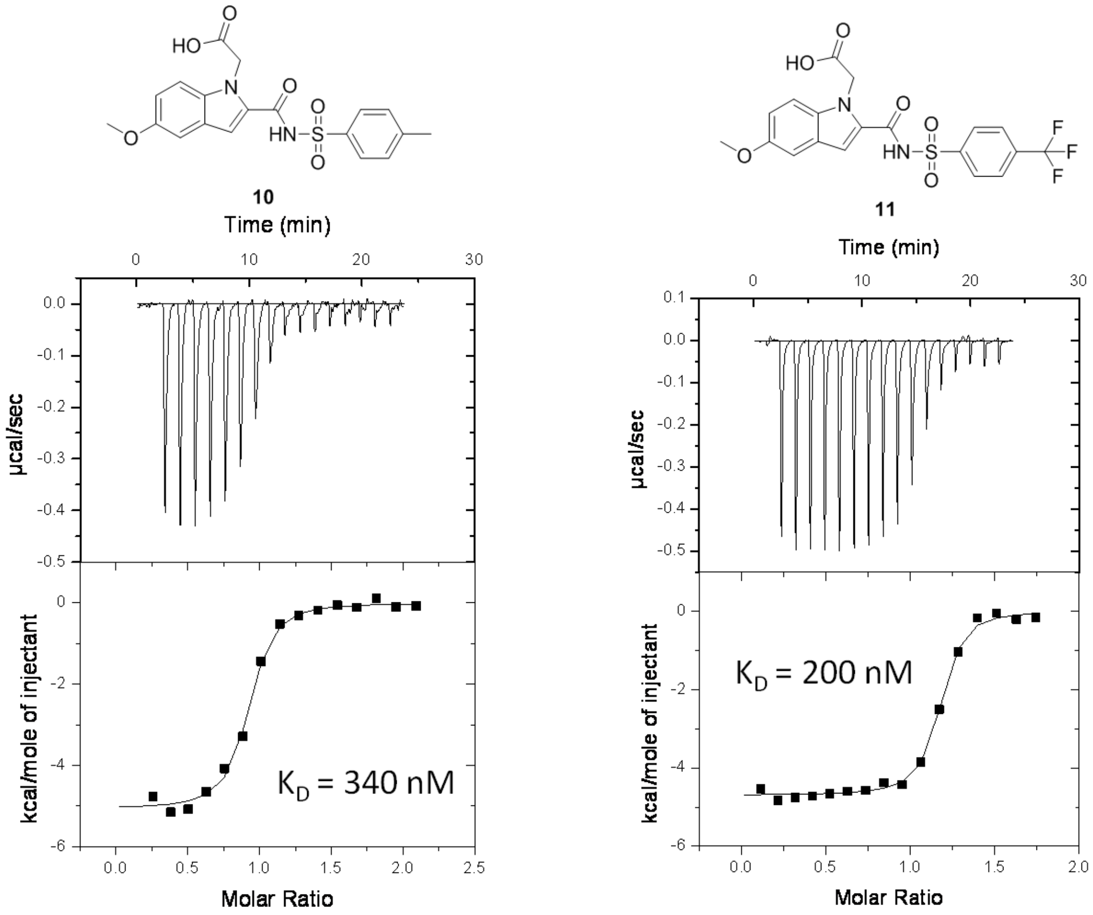

Figure S7. ITC titrations for **10** and **11** against *M.tuberculosis* pantothenate synthetase.

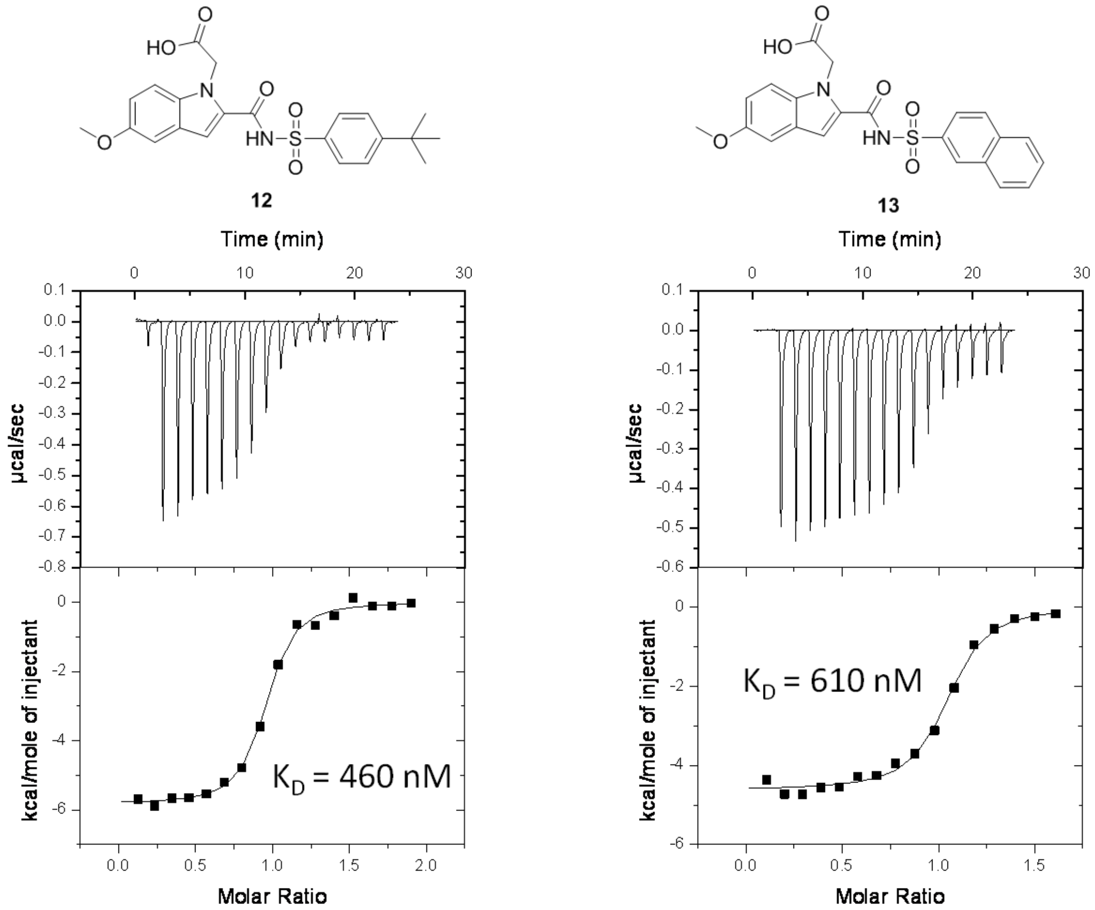

Figure S8. ITC titrations for **12** and **13** against *M.tuberculosis* pantothenate synthetase.

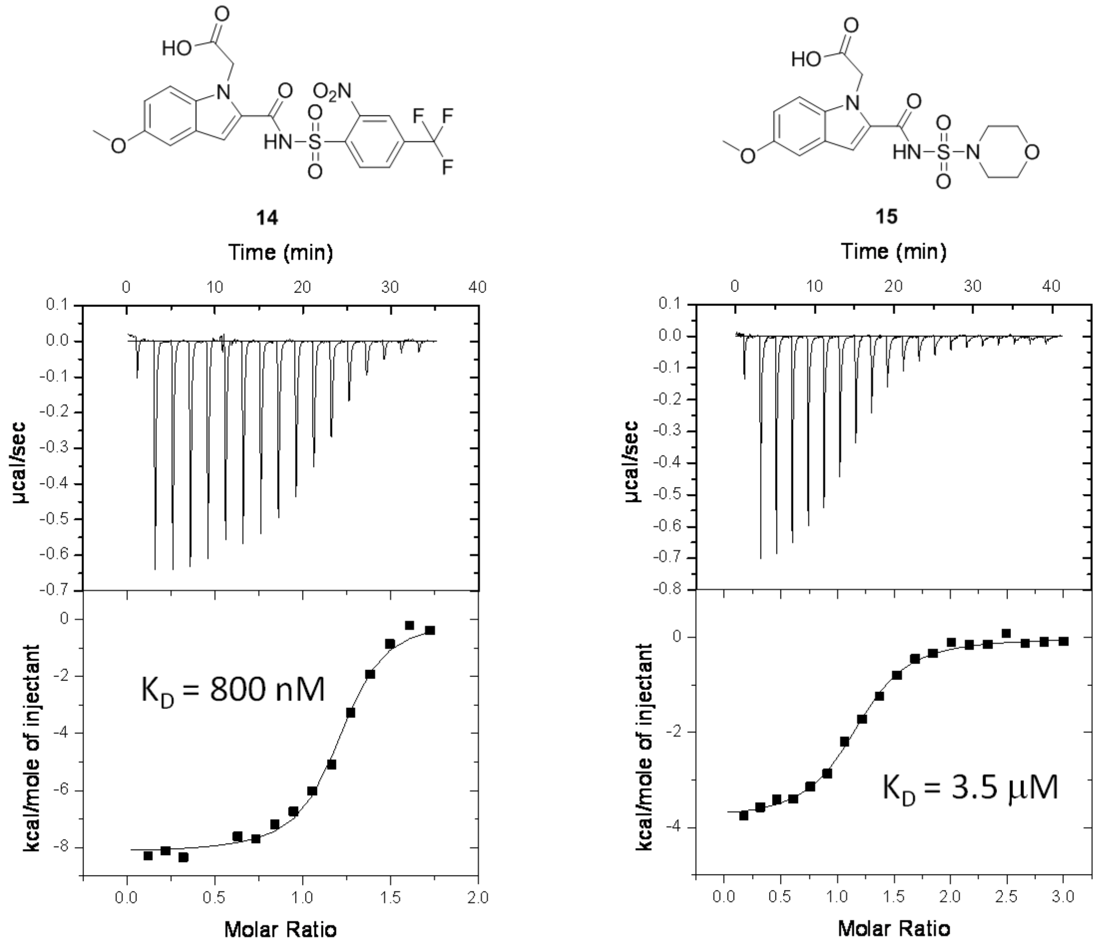

Figure S9. ITC titrations for **14** and **15** against *M.tuberculosis* pantothenate synthetase.

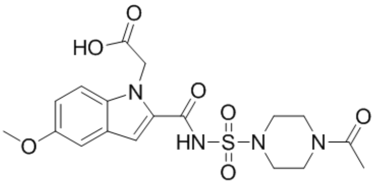

16

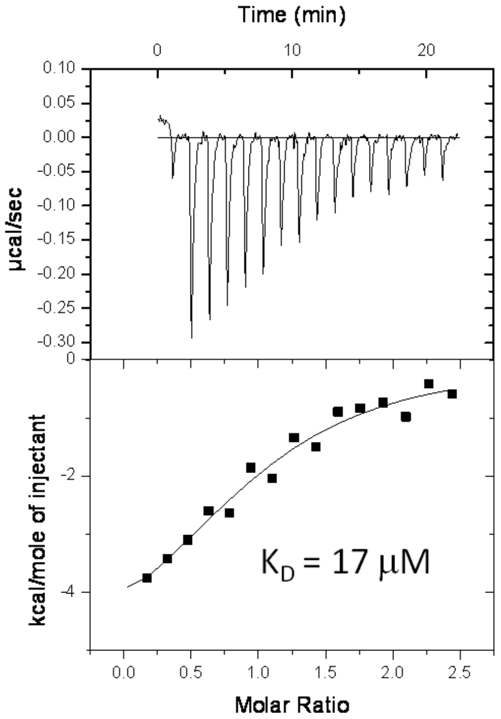

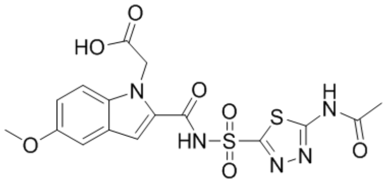

17

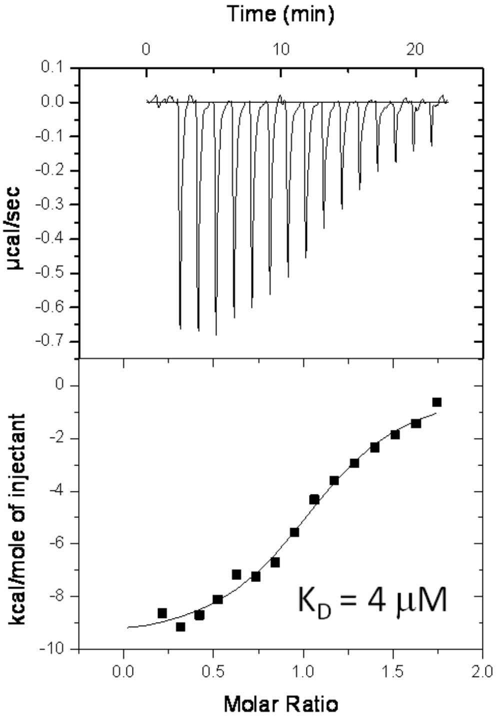

Figure S10. ITC titrations for **16** and **17** against *M.tuberculosis* pantothenate synthetase.

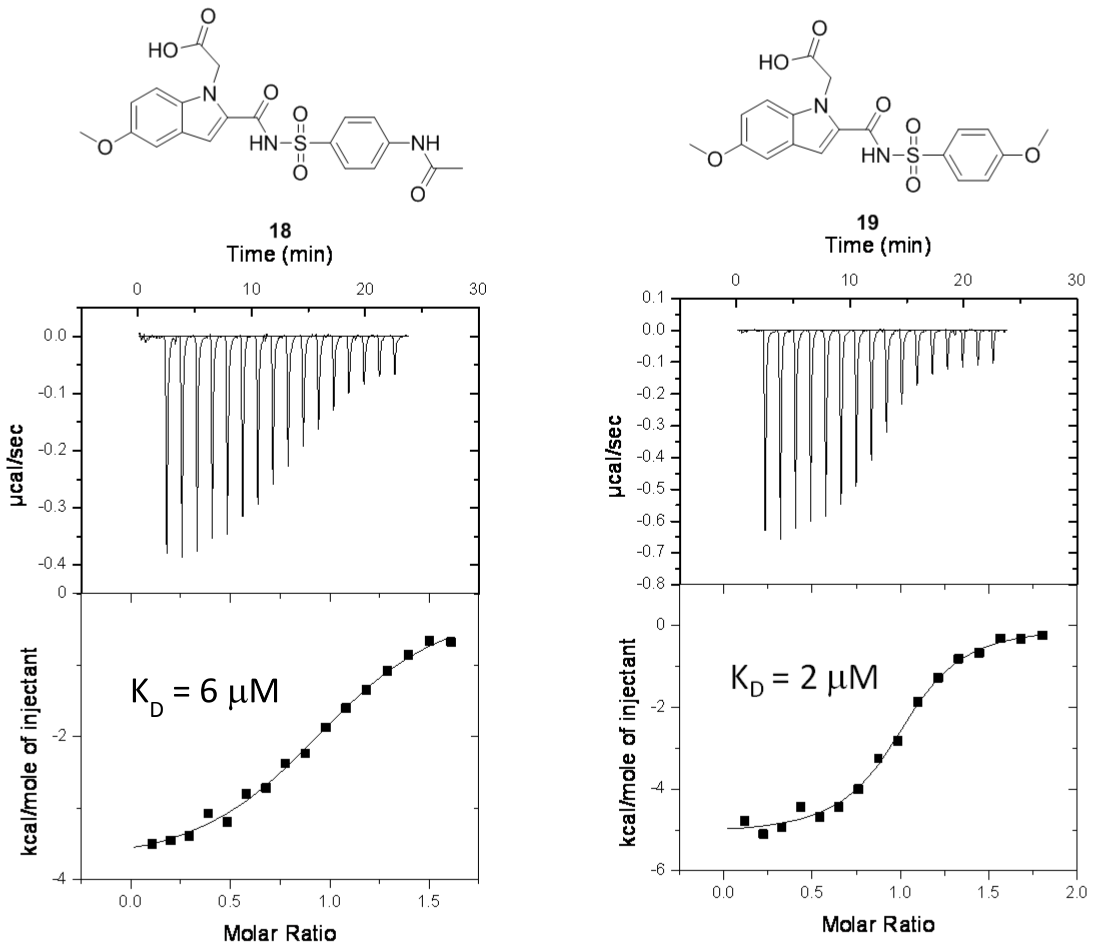

Figure S11. ITC titrations for **18** and **19** against *M.tuberculosis* pantothenate synthetase.

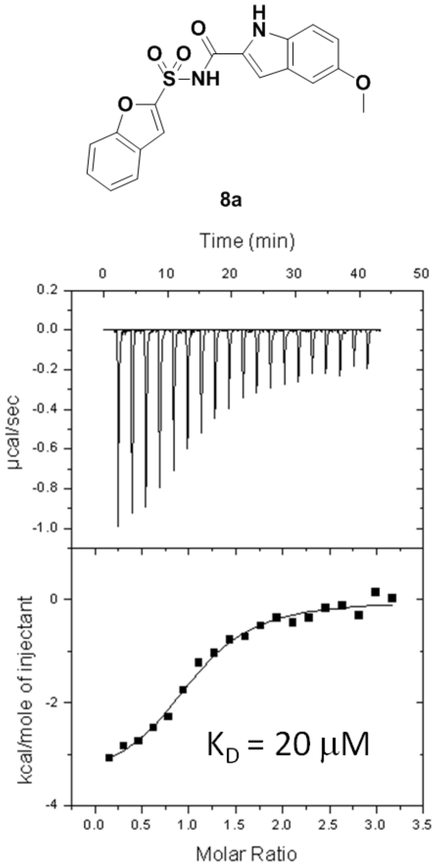

Figure S12. ITC titrations for **8a** against *M.tuberculosis* pantothenate synthetase.

Biochemical Assay[5] - Pantothenate synthetase activity was assayed by coupling formation of AMP to the oxidation of NADH with three other enzymes, myokinase, pyruvate kinase and lactate dehydrogenase as previously described[1]. The decrease in absorbance of NADH at 340 nM was measured in a 96-well Bio-Tek Powerwave XS plate reader at 25 ^{o}C. The assay contained 50 mM HEPES-HCl pH 7.6, 50 mM sodium chloride, 5 mM magnesium chloride**,** 1.5 mM potassium phosphoenolpyruvate, 100 nM His_6-PS, 0.2 mM NADH, 5 units of pyruvate kinase, 5 units of myokinase, 6 units of lactate dehydrogenase and 4 mM β-alanine. ATP and inhibitor (in DMSO making up 10 % v/v well concentration) concentrations were varied throughout the experiments as indicated. 4 mM Pantoate was used to initiate the enzymatic reaction**.** The rate of the pantothenate synthetase reaction was determined by the following equation:

$$\text{Rate}/\mu\text{mol s}^{-1} = 10^{6} \text{ x } A_{340} / 2 \text{ x } \varepsilon_{340} \text{ x l x time}$$

A_{340} is the absorbance of at 340nM

l = 0.54 cm^{-1} for 200 μl volume in each of the well

ε_{340} = 6220 M^{-1} cm^{-1} for NADH

The assay was performed in duplicates and least-squares fitting of the rate of the reaction to the Michaelis-Menten and Lineweaver-Burk equations were determined using the GraFit software (version 5.0.6, Erithacus Software Limited).

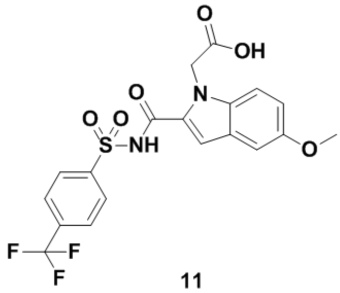

11
IC$_{50}$ = 5.7 µM

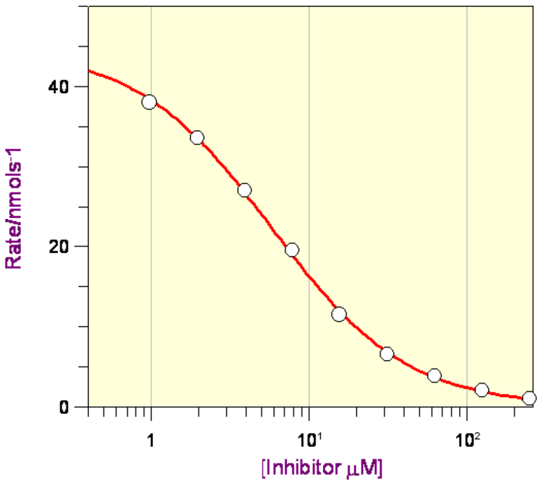

Parameter	Value	Std. Error
IC 50	5.7	0.1

Inhibition data against pantothenate synthetase. Compound **11** displayed an IC$_{50}$ of 5.7 µM.

Synthesis

General Experimental Section. All reactions were performed under nitrogen. Column chromatography was performed using silca gel (230 – 400 mesh). ^{1}H NMR spectra were recorded on either a Bruker DPX-400 MHz or a Bruker DPX-500 MHz spectrometer. High-resolution mass spectrometry (HRMS) was carried out using a Micromass Quadrupole-Time of flight spectrometer. Liquid-chromatography mass spectrometry (LCMS) was carried out using an Alliance HT Waters 2395 Separations Module coupled to a photomultiplier detection system. In LCMS, the first eluent is 10 mM ammonium acetate and the second eluent 95% aqueous acetonitrile. Samples were run on a gradient from 0-100% over a period of 8 min. Infrared (IR) spectroscopy of solids were recorded on a Perkin Elmer Spectrum One FTIR spectrometer using attenuated transmittance reflectance.

1-(2-*tert*-Butoxy-2-oxoethyl)-5-methoxy-1*H*-indole-2-carboxylic acid (1a)[1].

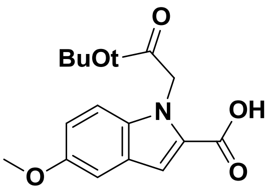

To a stirred solution of 5-methoxyindole-2-carboxylic acid (0.500 g, 2.60 mmol) in anhydrous DMF (10 ml) at 0 °C was added sodium hydride 60% in mineral oil (0.240 g, 6.03 mmol) over a 5 min period. The mixture was stirred for 1 h at 25 °C. To this mixture was added *tert*-butyl bromoacetate (0.43 mL, 2.9 mmol) in DMF (5 ml) at 0 °C and the mixture stirred for an additional 3 h at 25 °C. The mixture was cooled to 0 °C and aqueous 1 M HCl was added until pH 3-4. Saturated aqueous ammonium chloride (10 mL) was added to the reaction mixture and the aqueous phase extracted with ethyl acetate (4 x 15 mL). The combined extracts were dried over sodium sulfate and concentrated *in vacuo*. Purification by column chromatography (1:30 – 1:10 methanol/dichloromethane) gave the indole acid **1a** (0.70 g, 88%) as a white solid.

^{1}H NMR (400 MHz, d^6-acetone): δ 7.44 (d, J = 9.0 Hz, 1H), 7.29 (d, J =0.7 Hz, 1H), 7.19 (d, J = 2.4 Hz, 1H), 7.02 (dd, J = 2.4 Hz, J = 9.0 Hz, 1H), 5.28 (s, 2H), 3.84 (s, 3H), 1.45 (s, 9H).
^{13}C NMR (100 MHz, d^6-acetone): δ 168.3, 162.9, 155.6, 135.6, 128.4, 126.8, 116.8, 111.5, 110.5, 102.9, 81.4, 55.3, 47.0, 27.7;
IR, ν_{max} (ATR): 1524, 1667, 1739 cm^{-1}.
LCMS (*m/z*) 304.0 $[M-H]^-$, retention time 4.04 min.
HRMS (*m/z*) for $C_{16}H_{19}NO_5$ $[M+H]^+$, calcd: 306.1341, found: 306.1353.

2-(5-Methoxy-2-(tosylcarbamoyl)-1*H*-indol-1-yl)acetic acid (10)

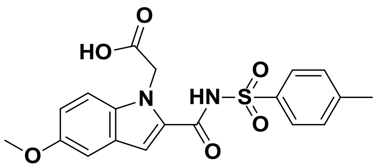

To a stirred solution of **1a** (0.070 g, 0.23 mmol) in dichloromethane (20 mL) was added EDCI (0.065 g, 0.34 mmol), DMAP (0.005 g, cat.), 4-methylbenzenesulfonamide (0.039 g, 0.23 mmol) and triethylamine (0.048 mL, 0.35 mmol). The mixture was stirred for 16 h at 25 °C. Saturated aqueous sodium bicarbonate (15 mL) was added to the reaction mixture and the aqueous phase extracted with dichloromethane (3 x 15 mL). The combined extracts were dried over sodium sulfate and concentrated *in vacuo*. Purification by column chromatography (gradient 0 -10% methanol/dichloromethane) gave the *tert*-butyl protected indole sulfonamide as a white solid (0.051 g, 49%).
To a solution of this *tert*-butyl protected indole (0.051 g, 0.11 mmol) in dichloromethane (3 mL) was added TFA (3 mL) dropwise. The mixture was stirred for 1 h at 25 °C and concentrated under *vacuo*. Saturated aqueous sodium bicarbonate (10 mL) was added to the residual solid, and the obtained aqueous solution was washed with ethyl acetate (2 x 10 mL). The aqueous phase was acidified with 1 M aqueous HCl until pH 2-3 and extracted with ethyl acetate (3 x 15 mL). The combined extracts were dried over sodium sulfate and concentrated *in vacuo*. The residual solid was triturated in ether (2 x 5 mL) to give **10** (0.034 g, 76%) as a white solid.

^{1}H NMR (500 MHz, d^4-methanol): δ 7.95 (d, J = 8.5 Hz, 2H), 7.38 (d, J = 8.5 Hz, 2H), 7.30 (s, 1H), 7.28 (d, J = 9.1, 1H), 7. 12 (d, J = 2.4 Hz, 1H), 6.85 (dd, J = 2.4 Hz, J = 9.1 Hz, 1H), 5.15 (s, 2H), 3.81 (s, 3H), 2.43 (s, 3H).
^{13}C NMR (125 MHz, d^4-methanol): δ 172.5, 161.8, 156.6, 145.9, 138.4, 136.9, 130.5 (x 2), 129.7, 129.2 (x 2), 127.6, 118.5, 112.0, 110.4, 103.7, 56.1, 46.9, 21.6.

IR, ν_{max} (ATR): 1692.8, 1522.7, 1404.6 cm^{-1}.
LCMS (MH^- 401.5), retention time 4.0 min.
HRMS (ES) for $C_{19}H_{19}N_2O_6S$ (MH^+) calcd: 403.0964, found: 403.0977.

2-(5-Methoxy-2-(4-(trifluoromethyl)phenylsulfonylcarbamoyl)-1*H*-indol-1-yl)acetic acid (11)

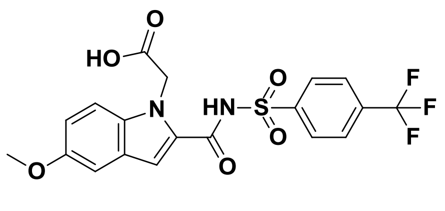

To a stirred solution of **1a** (0.055 g, 0.18 mmol) in dichloromethane (20 mL) was added EDCI (0.052 g, 0.27 mmol), DMAP (0.005 g, cat.), 4-trifluoromethyl benzenesulfonamide (0.041 g, 0.18 mmol) and triethylamine (0.038 mL, 0.27 mmol). The mixture was stirred for 6 h at 25 ^{o}C. Saturated aqueous sodium bicarbonate (15 mL) was added to the reaction mixture and the aqueous phase extracted with dichloromethane (3 x 15 mL). The combined extracts were dried over sodium sulfate and concentrated *in vacuo*. Purification by column chromatography (gradient 0 -10% methanol/dichloromethane) gave the *tert*-butyl protected indole sulfonamide as a white solid.

To a solution of this *tert*-butyl protected indole in dichloromethane (4 mL) was added TFA (4 mL) dropwise. The mixture was stirred for 45 min at 25 ^{o}C and concentrated under *vacuo*. Saturated aqueous sodium bicarbonate (10 mL) was added to the residual solid, and the obtained aqueous solution was washed with ethyl acetate (2 x 10 mL). The aqueous phase was acidified with 1 M aqueous HCl until pH 2-3 and extracted with ethyl acetate (3 x 15 mL). The combined extracts were dried over sodium sulfate and concentrated *in vacuo*. The residual solid was triturated in ether (2 x 5 mL) to give **11** (0.034 g, 41% after two steps) as a white solid.

^{1}H NMR (500 MHz, d^{6}-acetone): δ 8.31 (d, J = 8.5 Hz, 2H), 8.01 (d, J = 8.5 Hz, 2H), 7.52 (s, 1H), 7.48 (d, J = 9.1 Hz, 1H), 7.14 (d, J = 2.4 Hz, 1H), 7.02 (dd, J = 2.4 Hz, J = 9.1 Hz, 1H), 5.26 (s, 2H), 3.81 (s, 3H).
^{13}C NMR (125 MHz, d^{6}-acetone): δ 170.4, 160.8, 156.5, 150.0, 136.9, 135.5 (q, J = 32.6 Hz), 130.3, 129.1, 127.5, 126.0, 123.8, 118.8, 112.6, 110.7, 103.7, 56.2, 46.9.
IR, ν_{max} (ATR): 2970.8, 1524.1 cm^{-1}.
LCMS (MH^- 455.4), retention time 4.4 min.
HRMS (ES) for $C_{19}H_{16}N_2O_6SF_3$ (MH^+) calcd: 457.0681, found: 457.0687.

2-(2-(4-*tert*-Butylphenylsulfonylcarbamoyl)-5-methoxy-1*H*-indol-1-yl)acetic acid (12)

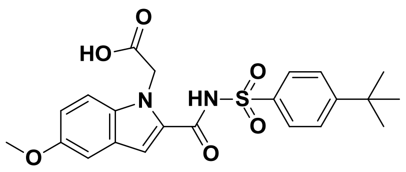

To a stirred solution of **1a** (0.060 g, 0.20 mmol) in dichloromethane (20 mL) was added EDCI (0.052 g, 0.30 mmol), DMAP (0.005 g, cat.), 4-*tert*butylbenzenesulfonamide (0.050 g, 0.23 mmol) and triethylamine (0.030 mL, 0.30 mmol). The mixture was stirred for 16 h at 25 ^{o}C. Saturated aqueous sodium bicarbonate (15 mL) was added to the reaction mixture and the aqueous phase extracted with dichloromethane (3 x 15 mL). The combined extracts were dried over sodium sulfate and concentrated *in vacuo*. Purification by column chromatography (gradient 0 -10% methanol/dichloromethane) gave the *tert*-butyl protected indole sulfonamide as a white solid.

To a solution of this *tert*-butyl protected indole in dichloromethane (4 mL) was added TFA (4 mL) dropwise. The mixture was stirred for 45 min at 25 ^{o}C and concentrated under *vacuo*. Saturated aqueous sodium bicarbonate (10 mL) was added to the residual solid, and the obtained aqueous solution was washed with ethyl acetate (2 x 10 mL). The aqueous phase was acidified with 1 M aqueous HCl until pH 2-3 and extracted with ethyl acetate (3 x 15 mL). The combined extracts were dried over sodium sulfate and concentrated *in vacuo*. The residual solid was triturated in ether (2 x 5 mL) to give **12** (0.010 g, 11% after two steps) as a white solid.

^{1}H NMR (500 MHz, d^{6}-acetone): δ 8.01 (d, J = 6.5 Hz, 2H), 7.66 (d, J = 6.5 Hz, 2H), 7.48 (s, 1H), 7.44 (d, J = 9.1 Hz, 1H), 7.13 (d, J = 2.4 Hz, 1H), 7.01 (dd, J =2.4 Hz, J = 9.1 Hz, 1H), 5.28 (s, 2H), 3.81 (s, 3H), 1.35 (s, 9H).

^{13}C NMR (125 MHz, d^{6}-acetone): δ 31.6, 36.2, 47.1, 56.2, 103.7, 110.2, 112.6, 118.4, 127.1, 127.5, 129.2, 136.8, 138.7, 156.5, 158.3, 161.0, 170.5.

IR, ν_{max} (ATR): 1773.4, 1664.2 cm^{-1}.

LCMS (MH^{-} 443.5), retention time 4.2 min.

HRMS (ES) for $C_{22}H_{25}N_2O_6S$ (MH^{+}) calcd: 445.1433, found: 445.1443.

2-(5-Methoxy-2-(naphthalen-2-ylsulfonylcarbamoyl)-1*H*-indol-1-yl)acetic acid (13)

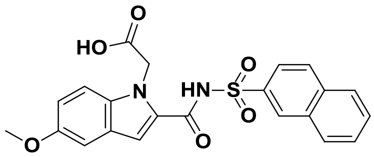

To a stirred solution of **1a** (0.100 g, 0.33 mmol) in dichloromethane (25 mL) was added EDCI (0.095 g, 0.495 mmol), DMAP (0.005 g, cat.), naphthalene-2-sulfonamide (0.082 g, 0.396 mmol) and triethylamine (0.068 mL, 0.491 mmol).The mixture was stirred for 16 h at 25 ^oC. Saturated aqueous sodium bicarbonate (15 mL) was added to the reaction mixture and the aqueous phase extracted with dichloromethane (3 x 15 mL). The combined extracts were dried over sodium sulfate and concentrated *in vacuo*. Purification by column chromatography (gradient 0 -10% methanol/dichloromethane) gave the *tert*-butyl protected indole sulfonamide as a solid.

To a solution of this *tert*-butyl protected indole in dichloromethane (4 mL) was added TFA (4 mL) dropwise. The mixture was stirred for 45 min at 25 ^oC and concentrated under *vacuo*. Saturated aqueous sodium bicarbonate (10 mL) was added to the residual solid, and the obtained aqueous solution was washed with ethyl acetate (2 x 10 mL). The aqueous phase was acidified with 1 M aqueous HCl until pH 2-3 and extracted with ethyl acetate (3 x 15 mL). The combined extracts were dried over sodium sulfate and concentrated *in vacuo*. The residual solid was triturated in ether (2 x 5 mL) to give **13** (0.011 g, 8% after two steps) as a white solid.

^{1}H NMR (500 MHz, d^6-acetone): δ 8.71 (m, 1H), 8.20 (d, J = 7.2 Hz, 1H), 8.14 – 8.02 (m, 3H), 7.74 – 7.61 (m, 2H), 7.52 (d, J = 0.7 Hz, 1H), 7.44 (d, J = 9.1 Hz, 1H), 7.13 (d, J = 2.4 Hz, 1H), 7.00 (dd, J = 9.1 Hz, J =2.4 Hz, 1H), 5.23 (s, 2H), 3.81 (s, 3H).
^{13}C NMR (125 MHz, d^6-acetone): δ 170.4, 160.9, 156.5, 138.4, 136.8, 136.5, 133.3, 131.0, 130.8, 130.5, 130.3, 129.5, 129.2, 128.9, 127.5, 124.2, 118.5, 112.5, 110.3, 103.7, 56.2, 46.9.
IR, ν_{max} (ATR): 1521.5, 1667.6 cm^{-1}.
LCMS (MH$^-$ 437.5), retention time 4.3 min.
HRMS (ES) for $C_{22}H_{19}N_2O_6S$ (MH$^+$) calcd: 439.0964, found: 439.0974

2-(5-Methoxy-2-(2-nitro-4-(trifluoromethyl)phenylsulfonylcarbamoyl)-1*H*-indol-1-yl)acetic acid (14)

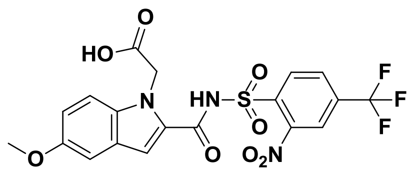

To a stirred solution of **1a** (0.150 g, 0.49 mmol) in dichloromethane (30 mL) was added EDCI (0.141 g, 0.74 mmol), DMAP (0.005 g, cat.), 2-nitro-4-(trifluoromethyl)benzenesulfonamide (0.159 g, 0.59 mmol) and triethylamine (0.10 mL, 0.72 mmol).The mixture was stirred for 7 h at 25 ^{o}C. Saturated aqueous sodium bicarbonate (15 mL) was added to the reaction mixture and the aqueous phase extracted with dichloromethane (3 x 15 mL). The combined extracts were dried over sodium sulfate and concentrated *in vacuo*. Purification by column chromatography (gradient 0 -10% methanol/dichloromethane) gave the *tert*-butyl protected indole sulfonamide as a white solid. To a solution of this *tert*-butyl protected indole in dichloromethane (4 mL) was added TFA (4 mL) dropwise. The mixture was stirred for 45 min at 25 ^{o}C and concentrated under *vacuo*. Saturated aqueous sodium bicarbonate (10 mL) was added to the residual solid, and the obtained aqueous solution was washed with ethyl acetate (2 x 10 mL). The aqueous phase was acidified with 1 M aqueous HCl until pH 2-3 and extracted with ethyl acetate (3 x 15 mL). The combined extracts were dried over sodium sulfate and concentrated *in vacuo*. The residual solid was triturated in methanol (2 x 2 mL) to give **14** (0.025 g, 10% after two steps) as a white solid.

^{1}H NMR (500 MHz, d^{6}-acetone): δ 11.35 (s, 1H), 8.64 (d, J = 8.3 Hz, 1H), 8.41 (s, 1H), 8.34 (d, J = 8.3 Hz, 1H), 7.70 (s, 1H), 7.50 (d, J = 9.1 Hz, 1H), 7.18 (d, J = 2.4 Hz, 1H), 7.05 (dd, J = 2.4 Hz, J = 9.1 Hz, 1H), 5.27 (s, 2H), 3.83 (s, 3H).
^{13}C NMR (125 MHz, d^{6}-acetone): δ 170.4, 160.9, 156.6, 149.8, 137.1, 136.7, 135.9, 130.4, 128.8, 127.5, 124.8, 123.5, 123.5, 119.1, 112.7, 111.5, 103.7, 56.2, 47.0.
IR, ν_{max} (ATR): 1683.7, 1556.0, 1523.6, 1359.6 cm^{-1}.
LCMS (MH^{-} 500.4), retention time 4.5 min.
HRMS (ES) for $C_{19}H_{15}N_3O_8SF_3$ (MH^{+}) calcd: 502.0532, found: 502.0535.

2-(5-Methoxy-2-(morpholinosulfonylcarbamoyl)-1*H*-indol-1-yl)acetic acid (15)

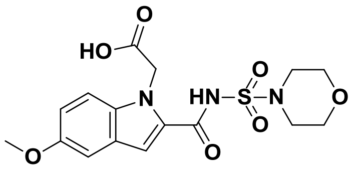

To a stirred solution of **1a** (0.150 g, 0.49 mmol) in dichloromethane (25 mL) was added EDCI (0.141 g, 0.74 mmol), DMAP (0.005 g, cat.), morpholine-4-sulfonamide (0.082 g, 0.49 mmol) and triethylamine (0.100 mL, 0.72 mmol). The mixture was stirred for 6 h at 25 ^{o}C. Saturated aqueous sodium bicarbonate (15 mL) was added to the reaction mixture and the aqueous phase extracted with dichloromethane (3 x 15 mL). The combined extracts were dried over sodium sulfate and concentrated *in vacuo*. Purification by column chromatography (1:1 ethyl acetate/hexane) gave the *tert*-butyl protected indole sulfonamide (0.051 g, 23%) as a white solid.

To a solution of this *tert*-butyl protected indole (0.025 g, 0.06 mmol) in dichloromethane (4 mL) was added TFA (4 mL) dropwise. The mixture was stirred for 1 h at 25 ^{o}C and concentrated under *vacuo*. Saturated aqueous sodium bicarbonate (10 mL) was added to the residual solid, and the obtained aqueous solution was washed with ethyl acetate (2 x 10 mL). The aqueous phase was acidified with 1 M aqueous HCl until pH 2-3 and extracted with ethyl acetate (3 x 15 mL). The combined extracts were dried over sodium sulfate and concentrated *in vacuo*. The residual solid was triturated both ether (1 x 2mL) and ethyl acetate (1 x 2 mL) to give **15** (0.017 g, 78%) as a off-white solid.

^{1}H NMR (500 MHz, d^{6}-acetone): δ 10.47 (s, 1H), 7.53 (d, J = 9.1 Hz, 1H), 7.46 (s, 1H), 7.14 (d, J = 2.4 Hz, 1H), 7.03 (dd, J = 2.4 Hz, J = 9.1 Hz, 1H), 5.39 (s, 2H), 3.82 (s, 3H), 3.69 (t, J = 4.9 Hz, 4H), 3.36 (t, J = 4.9 Hz, 4H).
^{13}C NMR (125 MHz, d^{6}-acetone): δ 170.8, 161.6, 156.5, 136.6, 129.8, 127.5, 118.2, 112.5, 109.7, 103.7, 67.3, 56.2, 47.9, 47.0.
IR, ν_{max} (ATR): 2901.4, 1771.7, 1670.4 cm^{-1}.
LCMS (MH^{-} 396.5), retention time 3.4 min.
HRMS (ES) for $C_{16}H_{20}N_3O_7S$ (MH^{+}) calcd: 398.1022, found: 398.0140.

2-(2-(4-Acetylpiperazin-1-ylsulfonylcarbamoyl)-5-methoxy-1*H*-indol-1-yl)acetic acid (16)

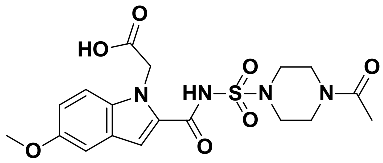

To a stirred solution of **1a** (0.045 g, 0.15 mmol) in dichloromethane (20 mL) was added EDCI (0.042 g, 0.22 mmol), DMAP (0.005 g, cat.), 4-acetylpiperazine-1-sulfonamide (0.034 g, 0.16 mmol) and triethylamine (0.031 mL, 0.22 mmol). The mixture was stirred for 6 h at 25 ^{o}C. Water (15 mL) was added to the reaction mixture and the aqueous phase extracted with dichloromethane (3 x 15 mL). The combined extracts were dried over sodium sulfate and concentrated *in vacuo*. Purification by column chromatography (1:9 methanol/ dichloromethane) gave the *tert*-butyl protected indole sulfonamide (0.019 g, 26%) as a white solid.

To a solution of this *tert*-butyl protected indole (0.019 g, 0.04 mmol) in dichloromethane (4 mL) was added TFA (4 mL) dropwise. The mixture was stirred for 1 h at 25 ^{o}C and concentrated under *vacuo*. Saturated aqueous sodium bicarbonate (10 mL) was added to the residual solid, and the obtained aqueous solution was washed with ethyl acetate (2 x 10 mL). The aqueous phase was acidified with 1 M aqueous HCl until pH 2-3 and extracted with ethyl acetate (3 x 15 mL). The combined extracts were dried over sodium sulfate and concentrated *in vacuo*. The residual solid was triturated with ether (2 x 2 mL) to give **16** (0.017 g, 61%) as a white solid.

^{1}H NMR (500 MHz, d^{4}-methanol): δ 7.36 (d, J = 9.1 Hz, 1H), 7.29 (s, 1H), 7. 13 (d, J = 2.4 Hz, 1H), 7.01 (dd, J = 2.4 Hz, J = 9.1 Hz, 1H), 5.37 (s, 2H), 3.82 (s, 3H), 3.63 (m, 4H), 3.44 (m, 4H), 2.10 (s, 3H)
^{13}C NMR (125 MHz, d^{4}-methanol): δ 172.7, 172.0, 162.4, 156.6, 136.7, 129.7, 127.7, 118.4, 111.9, 110.0, 103.7, 56.1, 47.7, 47.2, 47.1, 47.0, 42.3, 21.2.
IR, ν_{max} (ATR): 2901.2, 1695.4, 1672.6 cm^{-1}.
LCMS (MH^{-} 437.5), retention time 3.8 min.
HRMS (ES) for $C_{18}H_{23}N_4O_7S$ (MH^{+}) calcd: 439.1287, found: 439.1303.

2-(2-(5-Acetamido-1,3,4-thiadiazol-2-ylsulfonylcarbamoyl)-5-methoxy-1*H*-indol-1-yl)acetic acid (17)

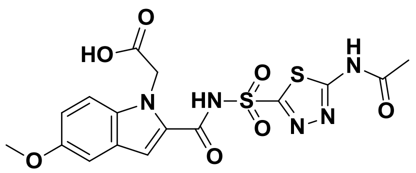

To a stirred solution of **1a** (0.170 g, 0.56 mmol) in dichloromethane (25 mL) was added EDCI (0.159 g, 0.83 mmol), DMAP (0.005 g, cat.), *N*-(5-sulfamoyl-1,3,4-thiadiazol-2-yl)acetamide (0.125 g, 0.56 mmol) and triethylamine (0.116 mL, 0.83 mmol). The mixture was stirred for 4 h at 25 ^{o}C. Water (15 mL) was added to the reaction mixture and the aqueous phase extracted with dichloromethane (3 x 15 mL). The combined extracts were dried over sodium sulfate and concentrated *in vacuo*. Purification by column chromatography (gradient 2 -10% methanol/dichloromethane) gave the *tert*-butyl protected indole sulfonamide (0.058 g, 20%) as a white solid.

To a solution of this *tert*-butyl protected indole (0.024 g, 0.05 mmol) in dichloromethane (3 mL) was added TFA (3 mL) dropwise. The mixture was stirred for 1 h at 25 ^{o}C and concentrated under *vacuo*. Saturated aqueous sodium bicarbonate (10 mL) was added to the residual solid, and the obtained aqueous solution was washed with ethyl acetate (2 x 10 mL). The aqueous phase was acidified with 1 M aqueous HCl until pH 2-3 and extracted with ethyl acetate (3 x 15 mL). The combined extracts were dried over sodium sulfate and concentrated *in vacuo*. The residual solid was triturated in ether (2 x 2 mL) to give **17** (0.012 g, 56%) as a pale yellow solid.

^{1}H NMR (500 MHz, d^{6}-acetone): δ 11.81 (s, 1H), 7.51 (s, 1H), 7.45 (d, J = 9.1 Hz, 1H), 7.15 (d, J = 2.4 Hz, 1H), 7.02 (dd, J = 2.4 Hz, J = 9.1 Hz, 1H), 5.30 (s, 2H), 3.82 (s, 3H), 2.37 (s, 3H).
^{13}C NMR (125 MHz, d^{6}-acetone): δ 170.4, 170.2, 156.5, 136.9, 127.6, 118.5, 112.6, 111.0, 103.7, 56.2, 47.3, 23.0.
IR, ν_{max} (ATR): 2902.5, 1699.0 cm^{-1}.
LCMS (MH^{-} 452.4), retention time 4.0 min.
HRMS (ES) for $C_{16}H_{15}N_5O_7S$ (MH^{+}) calcd: 454.0498, found: 454.0491.

2-(2-(4-Acetamidophenylsulfonylcarbamoyl)-5-methoxy-1*H*-indol-1-yl)acetic acid (18)

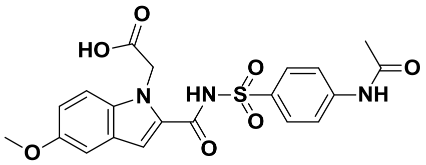

To a stirred solution of **1a** (0.055 g, 0.18 mmol) in dichloromethane (20 mL) was added EDCI (0.051 g, 0.27 mmol), DMAP (0.005 g, cat.), *N*-(4-sulfamoylphenyl)acetamide (0.046 g, 0.22 mmol) and triethylamine (0.037 mL, 0.27 mmol). The mixture was stirred for 16 h at 25 ^{o}C. Saturated aqueous sodium bicarbonate (15 mL) was added to the reaction mixture and the aqueous phase extracted with dichloromethane (3 x 15 mL). The combined extracts were dried over sodium sulfate and concentrated *in vacuo*. Purification by column chromatography (gradient 0 -10% methanol/dichloromethane) gave the *tert*-butyl protected indole sulfonamide as a solid.

To a solution of this *tert*-butyl protected indole in dichloromethane (4 mL) was added TFA (4 mL) dropwise. The mixture was stirred for 1 h at 25 ^{o}C and concentrated under *vacuo*. Saturated aqueous sodium bicarbonate (10 mL) was added to the residual solid, and the obtained aqueous solution was washed with ethyl acetate (2 x 10 mL). The aqueous phase was acidified with 1 M aqueous HCl until pH 2-3 and extracted with ethyl acetate (3 x 15 mL). The combined extracts were dried over sodium sulfate and concentrated *in vacuo*. The residual solid was triturated in ether (2 x 5 mL) to give **18** (0.020 g, 25% after two steps) as a white solid.

^{1}H NMR (500 MHz, d^{4}-methanol): δ 7.96 (d, J = 8.7 Hz, 2H), 7.74 (d, J = 8.7 Hz, 2H), 7.31 (d, J = 9.1 Hz, 1H), 7.27 (s, 1H), 7. 08 (d, J = 2.4 Hz, 1H), 6.94 (dd, J = 2.4 Hz, J = 9.1 Hz, 1H), 5.14 (s, 2H), 3.76 (s, 3H), 2.10 (s, 3H).
^{13}C NMR (125 MHz, d^{4}-methanol): δ 171.7, 171.5, 156.3, 144.8, 136.7, 130.3 (x 2), 127.4, 119.7 (x 2), 118.2, 112.2, 110.1, 103.6, 100.7, 97.7, 56.0, 46.9, 24.1.
IR, ν_{max} (ATR): 2901.4, 1666.4 cm^{-1}.
LCMS (MH^{-} 444.5), retention time 3.4 min.
HRMS (ES) for $C_{20}H_{20}N_3O_7S$ (MH^{+}) calcd: 446.0122, found: 446.1044.

2-(5-Methoxy-2-(4-methoxyphenylsulfonylcarbamoyl)-1*H*-indol-1-yl)acetic acid (19)

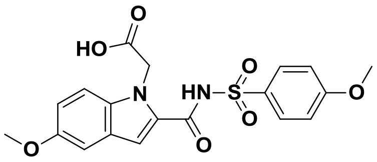

To a stirred solution of **1a** (0.030 g, 0.10 mmol) in dichloromethane (20 mL) was added EDCI (0.028 g, 0.15 mmol), DMAP (0.005 g, cat.), 4-methoxybenzenesulfonamide (0.022 g, 0.12 mmol) and triethylamine (0.021 mL, 0.15 mmol). The mixture was stirred for 6 h at 25 ^{o}C. Saturated aqueous sodium bicarbonate (15 mL) was added to the reaction mixture and the aqueous phase extracted with dichloromethane (3 x 15 mL). The combined extracts were dried over sodium sulfate and concentrated *in vacuo*. Purification by column chromatography (gradient 0 -10% methanol/dichloromethane) gave the *tert*-butyl protected indole sulfonamide as a solid.

To a solution of this *tert*-butyl protected indole in dichloromethane (4 mL) was added TFA (4 mL) dropwise. The mixture was stirred for 1 h at 25 ^{o}C and concentrated under *vacuo*. Saturated aqueous sodium bicarbonate (10 mL) was added to the residual solid, and the obtained aqueous solution was washed with ethyl acetate (2 x 10 mL). The aqueous phase was acidified with 1 M aqueous HCl until pH 2-3 and extracted with ethyl acetate (3 x 15 mL). The combined extracts were dried over sodium sulfate and concentrated *in vacuo*. The residual solid was triturated in ether (2 x 5 mL) to give **19** (0.011 g, 27% after two steps) as a white solid.

^{1}H NMR (500 MHz, d^{6}-acetone): δ 8.02 (d, J = 7.0 Hz, 2H), 7.48 (s, 1H), 7.45 (d, J = 9.1 Hz, 1H), 7.13 – 7.10 (m, 3H), 7.01 (dd, J = 2.4 Hz, J = 9.1 Hz, 1H), 5.28 (s, 2H), 3.91 (s, 3H), 3.81 (s, 3H).
^{13}C NMR (125 MHz, d^{6}-acetone): δ 170.5, 165.0, 160.8, 156.5, 136.8, 132.8, 129.7, 127.5, 118.4, 115.2, 113.3, 112.6, 112.5, 110.1, 110.0, 103.7, 56.5, 56.2, 46.9.
IR, ν_{max} (ATR): 3296.1, 1664.8 cm^{-1}.
LCMS (MH^{-} 417.5), retention time 3.7 min.
HRMS (ES) for $C_{19}H_{20}N_2O_7S$ (MH^{+}) calcd: 419.0913, found: 419.0911.

2-(5-Methoxy-2-(4-(trifluoromethyl)benzylsulfonylcarbamoyl)-1*H*-indol-1-yl)acetic acid (20)

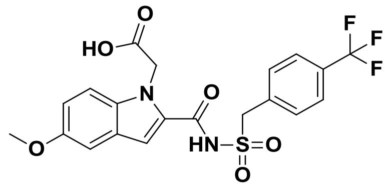

To a stirred solution of **1a** (0.044 g, 0.14 mmol) in dichloromethane (20 mL) was added EDCI (0.052 g, 0.27 mmol), DMAP (0.005 g, cat.), benzylsulfonoamide (0.035 g, 0.15 mmol) and triethylamine (0.039 mL, 0.28 mmol).The mixture was stirred for 16 h at 25 ^{o}C. Water (15 mL) was added to the reaction mixture and the aqueous phase extracted with dichloromethane (3 x 15 mL). The combined extracts were dried over sodium sulfate and concentrated *in vacuo*. Purification by column chromatography (gradient 0 -10% methanol/dichloromethane) gave the *tert*-butyl protected indole sulfonamide as a white solid. To a solution of this *tert*-butyl protected indole in dichloromethane (2 mL) was added TFA (4 mL) dropwise. The mixture was stirred for 1 h at 25 ^{o}C and concentrated under *vacuo*. Saturated aqueous sodium bicarbonate (10 mL) was added to the residual solid, and the obtained aqueous solution was washed with ethyl acetate (2 x 10 mL). The aqueous phase was acidified with 1 M aqueous HCl until pH 2-3 and extracted with ethyl acetate (3 x 15 mL). The combined extracts were dried over sodium sulfate and concentrated *in vacuo* to give **20** (0.011 g, 16% after two steps) as a white solid.

^{1}H NMR (500 MHz, d^{6}-acetone): δ 7.70 (d, J = 8.2 Hz, 2H), 7.65 (d, J = 8.2 Hz, 2H), 7.56 (d, J = 9.1 Hz, 1H), 7.42 (s, 1H), 7.12 (d, J = 2.4 Hz, 1H), 7.06 (dd, J = 9.1 Hz, J = 2.4 Hz, 1H), 5.47 (s, 2H), 4.96 (s, 2H), 3.81 (s, 3H).
^{13}C NMR (125 MHz, d^{6}-acetone): δ 170.9, 162.3, 156.5, 137.0, 135.2, 133.1 (x 2), 132.9, 129.2, 127.4, 126.7, 126.7, 118.8, 112.6, 110.9, 103.7, 59.2, 56.2, 47.1.
IR, ν_{max} (ATR): 2964.3, 1670.8 cm^{-1}.
LCMS (MH^{-} 469.5), retention time 4.2 min.
HRMS (ES) for $C_{20}H_{18}N_2O_6SF_3$ (MH^{+}) calcd: 471.0835, found: 471.0845

References

(1) Hung, A. W.; Silvestre, H. L.; Wen, S.; Ciulli, A.; Blundell, T. L.; Abell, C. . *Angew. Chem. Int. Ed.* **2009**, *48*, 8452.
(2) Sledz, P.; Silvestre, H. L.; Hung, A. W.; Ciulli, A.; Blundell, T. L.; Abell, C. *J. Am. Chem. Soc.* **2010**, *132*, 4544.
(3) G. N. Murshudov, A. A. Vagin, E. J. Dodson, *Acta Crystallogr. Sect. D* **1997**, *53,* 240.
(4) P. Emsley, K. Cowtan, *Acta Crystallogr. Sect. D* **2004**, *60*, 2126.
(5) Zheng, R.; Blanchard, J. S. *Biochemistry* **2001**, *40*, 12904.
